# Directly Gel‐Thermal Processing of Linker‐Mixed Crystal‐Glass Composite Membranes for Sorption‐Preferential Gas Separation

**DOI:** 10.1002/advs.202413942

**Published:** 2024-12-12

**Authors:** Yihao Xiao, Yanqing Yu, Xinxi Huang, Da Chen, Wanbin Li

**Affiliations:** ^1^ College of Environment and Climate Jinan University No. 855, East Xingye Avenue, Panyu District Guangzhou 511443 China

**Keywords:** gas separations, linker mixing, membranes, metal–organic frameworks, sol–gel

## Abstract

Membrane processes are promising for energy‐saving industrial applications. However, efficient separation for some valuable gas mixtures with similar characteristics, such as CH_4_/N_2_ and O_2_/N_2_, remains extremely challenging. Metal–organic framework (MOF) membranes have been attracting intensive attention for gas sieving, but it is difficult to manufacture MOF membranes in scalability and precisely tune their transport property. In this study, Gel‐thermal processing of linker‐mixed MOF crystal‐glass composite membranes is reported directly, with the mechanism of adjusting metal‐linker bond strengths and angles to disrupt long‐range periodicity of MOFs and promote glass phase formation, for sharply sorption‐preferential gas separation. This strategy can be realized by using a simple, solvent/precursor‐less, and cost‐effective gel‐thermal approach with two steps of gel coating and thermal conversion, thereby constructing crystal‐glass composite membranes in a controllable, processable, versatile, and environmentally friendly route. Moreover, the mixed linkers enable preferential gas affinities and the ultramicroporous glasses can eliminate any membrane defects. The membranes exhibit outstanding gas separation performance for the challenging systems of CH_4_/N_2_ and O_2_/N_2_, with mixture selectivities up to 9.3 and 9.6, respectively, far exceeding those of polymer, MOF, and mixed‐matrix membranes. The study provides an available route for architecting high‐performance membranes for gas separations.

## Graphical abstract

Linker‐mixed metal–organic framework crystal‐glass composite membranes are designed through directly gel‐thermal processing in a simple and scalable route, with the mechanism of adjusting metal‐linker bond features to disrupt long‐range periodicity of frameworks. Because mixed linkers enable preferential gas sorption properties and glasses suppress membrane defects, the prepared membranes have impressive CH_4_/N_2_ and O_2_/N_2_ separation performance and outperformed most other membranes.

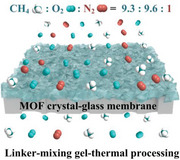

## 1 Introduction

Chemical separations, mainly involving heat‐driven processes, for example, distillation, account for half of the total industrial energy consumption.^[^ ^1^ ^]^ Membrane technology with inherent merits of energy saving, efficiency, small footprint, and others has great potential for separating gases and liquids.^[^ ^1^, ^2^ ^]^ Theoretically, the energy consumption can be reduced to one‐tenth as that of energy‐intensive distillation once appropriate membrane methods are adopted.^[^ ^1^ ^]^ Highly processable polymers are widely used for membrane fabrication, but the membranes suffer from the trade‐off limitation between permeability and selectivity.^[^ ^3^ ^]^ Moreover, for some valuable gas mixtures with similar characteristics, such as CH_4_/N_2_ and O_2_/N_2_, which are important for chemical, medical, semiconductor, environmental, and energy‐related industries, the conventional processes, e.g., cryogenic distillation and adsorption, are extremely energy‐intensive, while the membrane processes usually show poor separation capability.

MOFs are a category of microporous crystals with regularly nanoporous structure, tunable chemical property, and specific gas affinity.^[^ ^4^ ^]^ Various MOFs have been heterogeneously crystallized on porous substrates to form molecular sieving membranes.^[^ ^5^ ^]^ However, the poor controllability of intrinsic nanopores at atomic level and the framework flexibility from linker rotation make the membranes difficult to precisely separate some mixtures with similar physicochemical properties,^[^ ^6^ ^]^ For example, CH_4_/N_2_ and O_2_/N_2_, which are vital to natural gas purification and oxygen enrichment that are important to chemical, medical, and energy‐related industries. Meanwhile, the issues of grain boundaries and pinhole defects resulting from intergrowth of crystals and shear force effect during preparation usually lead to the deterioration of selectivity.^[^ ^5a^ ^]^ In addition, the scalable manufacture and simplified fabrication are prerequisites for the practical and industrial applications. So far, their scalable and low‐cost production of MOF membranes remains extremely challenging, although various synthesis approaches, including hydro/solvothermal treatment,^[^ ^6^ ^]^ interfacial/contra‐diffusion synthesis,^[^ ^7^ ^]^ chemical vapor deposition,^[^ ^8^ ^]^ and electrochemical growth,^[^ ^9^ ^]^ have been proved with feasibility to prepare MOF membranes.

Amorphous MOFs with distinctive features have attracted increasing attention.^[^ ^2^, ^10^ ^]^ MOF glasses inherit metal‐linker connection and short‐range order but are of long‐range disorder. Benefited from their functionality, reactivity, ultramicroporosity, and mouldability, MOF glasses have been demonstrated with good efficiency for adsorption, separation, catalysis, optical, and energy‐related applications.^[^ ^10^, ^11^ ^]^ Amorphous MOF membranes are ideal candidates to overcome the issues of pinhole defects and uncontrollable passageways.^[^ ^11^ ^]^ Generally, MOF glass and MOF crystal‐glass composites are transformed from MOFs by melting‐quenching,^[^ ^11^ ^]^ through melting crystals to break metal‐linker connections and obtain liquids, and then quenching to recombine coordination bonds for achieving glasses. Nonetheless, the elevated temperature over 400 °C is usually required for melting crystals to obtain glasses and the vacuum/inert gas protection should be applied to prevent linkers from being decomposed. Moreover, the types of MOF glasses are limited since crystals are often decomposed prior to melting. In addition, the harsh and complicated procedures are unfavorable to the processability of MOF glass membranes and MOF crystal‐glass composite membranes, especially for those with polymer substrates that are high‐temperature sensitive but suitable for scalable production.^[^ ^5^, ^7^, ^8^ ^]^

We envisage the adjustment of metal‐linker coordination properties to disrupt long‐range periodicity for designing MOF crystal‐glass composites in a scalable manner at low temperature. A linker‐mixing concept based on gel‐thermal processing is proposed to directly synthesize MOF crystal‐glass composite membranes on various substrates. In contrast to conventional melt‐quenched method, this linker‐mixing gel‐thermal processing, implemented through simply coating and thermally treating precursor gels in a normal room atmosphere, is mild, controllable, processable, versatile, and environment‐friendly, with no need for massive solvent and precursors, harsh temperature, and inert gas protection. Furthermore, as the mixed linkers enhance preferential gas affinity and the glasses eliminate non‐selective transport channels, the MOF crystal‐glass composite membranes exhibit great performance for the challenging separations of CH_4_/N_2_ and O_2_/N_2_.

## 2 Results and Discussion

### 2.1 Gel‐Thermal Processing of Linker‐Mixed Crystal‐Glass Composites

**Figure 1:**
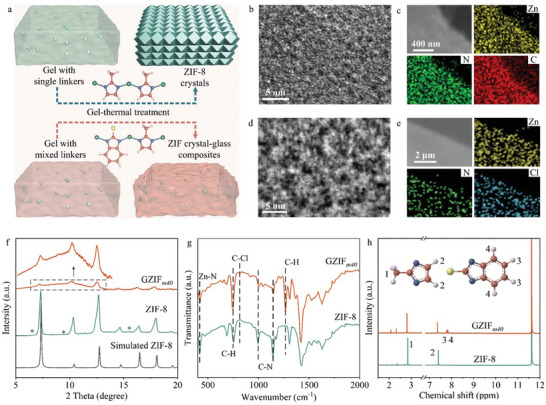
Gel‐thermal processing of ZIF‐8 and linker‐mixed GZIF*_m_*. a) Schematics of ZIF‐8 crystals and linker‐mixed ZIF crystal‐glass composites fabricated by gel‐thermal treatment. Zn, C, N, Cl, and H atoms are depicted in green, orange, blue, yellow, and grey, respectively. ZIF‐8 colloids in gels and composites are presented by green rhombic dodecahedrons. b) High‐resolution TEM image of ZIF‐8. c) Elemental distribution images of ZIF‐8 from EDX mapping. d) High‐resolution TEM image of GZIF*_m40_*. e) Elemental distribution images of GZIF*_m40_* from EDX mapping. f) XRD patterns of ZIF‐8 and GZIF*_m40_*. Inset present amplified XRD pattern of GZIF*_m40_*. Simulated XRD pattern of ZIF‐8 is presented for comparison. g) FTIR spectra of ZIF‐8 and GZIF*_m40_*. Some characteristic peaks are marked by dash lines. h) ^1^H NMR spectra of ZIF‐8 and GZIF*_m40_*. H positions and related characteristic peaks are marked by 1, 2, 3, and 4.

We processed the zeolitic imidazolate framework (ZIF) crystal‐glass composites with mixed 2‐methylimidazolate (MeIM) and 2‐chlorobenzimidazolate (ClBIM) linkers by gel‐thermal method (**Figure** **1**a; Figures S1, S2, Supporting Information). Several studies have proposed using linker‐mixed precursors to obtain MOF membranes,^[^ ^12^ ^]^ but those have not involved the construction of glasses and the gel‐thermal method. Because specific metal‐linker coordination properties facilitated glass formation and functional groups boosted preferential gas sorption, 2‐chlorobenzimidazole (HClBIM) was used here. Briefly, zinc acetate dihydrate as metal source and ethanolamine as chelating reagent were dispersed in ethanol to fabricate sol with layered basic zinc acetate nanocrystals and zinc‐ethanolamine complexes.^[^ ^8a^ ^]^ Then, the linker mixture was added to generate ZIF colloids and convert sol to viscous gel,^[^ ^13^ ^]^ which could promote precursor loading and membrane formation. Ultimately, the gel was diluted, spin‐coated, and thermally treated at a typical temperature of MOF crystallization (i.e., 120 °C) to volatilize solvent, additive, and excessive linker molecules for executing zinc‐linker reactions and achieving crystal‐glass composite membrane. During formation, the linkers were volatilized and substituted the organic segments of zinc‐based complexes simultaneously; while these organic components acquired protons from linkers and were removed. In this linker‐mixing gel‐thermal processing, the metal/linker molar ratio was 1:4, and the linker mixture had different HClBIM molar contents of 0%–60%. Higher HClBIM content (≥60%) caused non‐complete dissolution. Unlike the conventional synthesis methods of MOF membranes, which should take complex processes for precursor diffusion, substrate modification, heterogeneous nucleation, and crystallization into account, the synthesis procedures in this study were simple, controllable, and versatile, as all reactions were confined in a thin coated gel layer. As a result, the MOF crystal‐glass composite membranes could be deposited on various substrates.

To detect the internal structure by transmission electron microscopy (TEM), the linker‐mixed MOF crystal‐glass composites with HClBIM contents of 0%, 10%, and 40% were deposited on carbon films with copper mesh supports and labelled as GZIF*_m0_* (ZIF‐8), GZIF*_m10_*, and GZIF*_m40_*, respectively (Figure 1b–e; Figures S3, S4, Supporting Information). Clearly, the gel‐thermal treatment induced the formation of the continuous ZIF‐8 and GZIF*_m_* films. High‐resolution TEM image illustrated a typical ZIF‐8 sketch with uniform and loose dark zinc center spots and white cavity speckles (Figure 1b).^[^ ^14^ ^]^ No observable lattice was explained by possible destruction of high‐energy electron beam to frameworks.^[^ ^2^, ^14^ ^]^ For GZIF*_m40_*, a different TEM image as that of ZIF‐8 was captured, since TEM electrons were incoherently scattered in amorphous and glass materials (Figure 1d). Energy‐dispersive x‐ray spectroscopy (EDX) mapping confirmed the uniform element distributions in ZIF‐8 and GZIF*_m_* and the homogeneous chlorine in GZIF*_m_* (Figure 1c,e).

### 2.2 Chemical Characteristics of GZIF*_m_*

X‐ray diffraction (XRD) pattern demonstrated the well crystallinity of the ZIF‐8 film on a substrate (Figure S5, Supporting Information). As expected, the XRD peak intensity of GZIF*_m40_* was significantly weakened, implying the formation of massive amorphous phase. To mitigate the background effects, the films were scraped from substrates to obtain the powder samples. As shown in Figure 1f, the powder XRD peaks were intensive, but the peak positions and the peak intensity variations from ZIF‐8 to GZIF*_m_* were consistent for different samples. A small amount of layered basic zinc acetate from crystallization of zinc precursors in alkaline solution existed in ZIF‐8.^[^ ^8a^ ^]^ On the contrary, the XRD pattern of GZIF*_m40_* had rarely any peak of this crystal phase. This divergence might be ascribed to that the HClBIM (193 °C) with higher melting point than HMeIM (143 °C) and having functional groups changed the reaction environment, which might not be conducive to the formation of the XRD‐detectable basic zinc acetate crystals in GZIF*_m_*.

Fourier transform infrared (FTIR) data manifested a typical ZIF‐8 spectrum (Figure 1g). After linker mixing, the C‐Cl and phenyl C‐H peaks emerged in the FTIR spectrum of GZIF*_m_* and became strengthened as ClBIM increased (Figure 1g; Figure S6, Supporting Information). These results testified that ZIF‐8 and GZIF*_m_* were integrated through zinc‐imidazolate reaction and GZIF*_m_* were linker‐mixed. Different from the FTIR spectra of unreacted HMeIM and HClBIM with protons, the absence of the N‐H peaks in the FTIR spectra of ZIF‐8 and GZIF*_m_* verified the deprotonation property and coordination state of linkers, rather than physical loading (Figures S7, S8, Supporting Information). As demonstrated previously,^[^ ^13a^ ^]^ the HMeIM would be removed by thermal treatment. Almost complete volatilization of HClBIM at 120 °C within 24 h further corroborated that the linkers in GZIF*_m_* were integrated through coordination (Figure S9, Supporting Information). In line with the XRD results, the carboxylate and ‐OH peaks in FTIR of as‐synthesized ZIF‐8 and GZIF*_m_* suggested the existence of basic zinc acetate (Figures S6, S7, Supporting Information), which could be removed by thermal treatment as discussed below. X‐ray photoelectron spectroscopy (XPS) revealed the chlorine presence in GZIF*_m_* and its peak was intensified for GZIF*_m_* along with increasing HClBIM (Figure S10, Supporting Information). A new bond of C‐Cl occurred in C 1s XPS of GZIF*_m_* (Figure S11, Supporting Information). Because the different strengths of Zn‐ClBIM and Zn‐MeIM changed the electron cloud distribution, a slight shift appeared in the Zn 2p XPS spectra of GZIF*_m_* (Figure S12, Supporting Information).

To measure the actual linker ratio, the as‐synthesized GZIF*_m_* samples were analyzed by proton nuclear magnetic resonance (^1^H NMR). Accordingly, the ClBIM proportions of GZIF*_m10_*, GZIF*_m20_*, GZIF*_m40_*, and GZIF*_m60_* were determined as 18.9%, 33.0%, 37.1%, and 45.2%, respectively (Figure 1h; Figures S13, S14, Supporting Information). As expected, the as‐synthesized ZIF‐8 and GZIF*_m_* samples contained a small amount of basic zinc acetate, e.g., GZIF*_m40_* having acetate to linker ratio of 7.6%. We further thermally treated GZIF*_m10_* at 220 °C for 3 h to remove any possibly volatile solvents, linkers, segments, and other guests. This temperature could make the linkers completely volatilized within 5 min (Figure S9, Supporting Information). If GZIF*_m10_* contained unreacted linkers, the volatilization of linkers with different volatilities theoretically induced linker ratio variation. However, the linker proportions only slightly varied, substantiating almost no physically loaded linkers (as discussed in Supporting Information, Figure S15, Supporting Information). Notably, the acetate segments of basic zinc acetate were completely removed as well. Thermogravimetric analysis (TGA) profiles collected in air atmosphere illustrated the good thermal stability of ZIF‐8 and GZIF*_m_* (Figures S16–S18, Supporting Information). Zinc oxide residue for ZIF‐8 (41.3%) was more than the theoretical value (35.8%) and that of the solvothermally synthesized ZIF‐8 (34.8%), owing to the small amount of zinc‐based complexes. For GZIF*_m_*, the zinc oxide contents were less than that of the solvothermal ZIF‐8 and decreased with the increase of ClBIM. This was attributed to the larger molecular weight of ClBIM than MeIM. In addition, the GZIF*_m_* samples with more ClBIM had better affinity and could adsorb more guests, solvents, and additives that could be removed at relatively low temperature. Relative to ZIF‐8, the GZIF*_m_* composites were more thermally stable and had higher decomposition temperature in air atmosphere (Figure S16, Supporting Information). For example, the GZIF*_m40_* sample became decomposed at 550 °C and had a derivative TG peak at 647 °C (Figure S17, Supporting Information), which were higher than those for ZIF‐8 and other GZIF*_m_*.

### 2.3 Validation and Formation Mechanism

**Figure 2:**
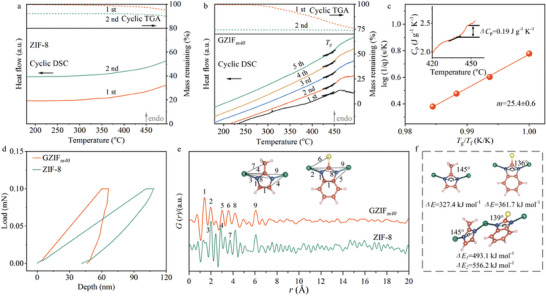
Calorimetry and structure of ZIF‐8 and GZIF*_m_*. a) Cyclic TGA (dash lines) and DSC curves (solid lines) of ZIF‐8. b) Cyclic TGA (dash line) and DSC curves (solid line) of GZIF*_m40_*. Different upscans with heating rate of 10 °C min^−1^ are presented by lines with different colors. Glass transition temperature (*T*_g_) is marked. c) Determination of fragility index (m) of GZIF*_m40_* obtained by cyclic DSC scans with different heating and cooling rates. *T_f_* was determined as *T_g_* with a heating rate of 10 °C min^−1^. Inset shows heat capacity (*C_p_*) curve obtained by 2nd DSC upscan. d) Load‐displacement curves of ZIF‐8 and GZIF*_m40_* obtained from nanoindentation. e) PDF profiles of ZIF‐8 and GZIF*_m40_* within the range of 0–20 Å. Insets show the Zn‐MeIM‐Zn and Zn‐ClBIM‐Zn structures and the corresponding distances between different atoms. Interatomic distances between different atoms are marked by Arabic numerals. f) Chemical structures and bond angles and energies of Zn‐MeIM‐Zn, Zn‐ClBIM‐Zn, and MeIM‐Zn‐ClBIM.

For validating the glass phase, the cyclic TGA of ZIF‐8 and GZIF*_m40_* were performed from room temperature to 500 °C (**Figure** **2**a,b) with N_2_ protection. In an inert atmosphere, the 1st TGA profile of GZIF*_m40_* showed weight loss due to the volatilization of adsorbed guests, acetates, and solvents, while almost no weight variation was observed in 2nd TGA curve. These results were also found in the thermal analyses of other MOF crystal‐glass composites.^[^ ^10^, ^11^ ^]^ After confirming the thermal stability by TGA in air and inert atmospheres, ZIF‐8 and GZIF*_m40_* were scanned by differential scanning calorimetry (DSC) in N_2_ atmosphere (Figure 2a,b; Figures S19, S20, Supporting Information). No endothermic peak was observed in the 1st upscan of ZIF‐8, because of its high stability, low absorptivity for guest molecules, and non‐melting property in the tested temperature range.^[^ ^15^ ^]^ An identical 2nd upscan attested the retained structures of ZIF‐8. Differently, GZIF*_m_* had two signals in the 1st DSC upscan curve, assigned to desorption and glass transition, respectively. The *T_g_* signal onset at 438 °C with peak‐like shape was resulted from glass transition accompanied by molecular relaxation, which usually existed in that of glasses after aging or with different thermal histories.^[^ ^16^ ^]^ After eliminating the thermal history, the 2nd upscan resembled 1st upscan and had a sigmoidal signal of glass transition at the same temperature, except for the absence of desorption peak. For melt‐quenched MOF glasses, the 1st upscan curve had two peaks of desorption and crystal‐liquid melting response, while the 2nd upscan curve only showed glass transition response.^[^ ^11^ ^]^ It was worth emphasizing that the glass transition signal in the 2nd upscan of melt‐quenched MOF glasses appeared at lower temperature than the melting point of MOFs and often had a similar peak‐like shape as the signal in 1st upscan of GZIF*_m40_*.^[^ ^10c,e,k^ ^]^ Notably, all DSC upscans of 2–5 after eliminating the thermal history displayed the identical sigmoidal *T_g_* signals (Figure 2b; Figure S20, Supporting Information). Another GZIF*_m40_* sample prepared under the same conditions proved the repeatability of calorimetric results from 5‐cycle heating‐cooling DSC (Figure S20, Supporting Information). These results evidenced that the signals in the DSC scans of GZIF*_m40_* were generated by glass transition, confirming that linker‐mixing gel‐thermal processing could directly prepare MOF crystal‐glass composites.

Besides TGA and DSC, the characterizations of heat capacity, fragility, and mechanical property were carried to investigate the features of MOF crystal‐glass composite. Through using the DSC scan of sapphire as a reference, the heat capacity change (*ΔC_p_*) from glass transition of GZIF*_m40_* was calculated as 0.19 J g^−1^ K^−1^ (Figure 2c, inset), which was similar to that of other MOF glasses (Table S1, Supporting Information). To study the fragility of GZIF*_m40_*, the cyclic DSC scans with a same heating rate as prior cooling rate were collected to monitor the temperature of glass transition at variable heating rates. Based on the temperature variation, the fragility index could be calculated as *m* = 25.4 (Figure 2c; Figure S21, Supporting Information). This value was comparable to that of silica and other MOF glasses (Table S1, Supporting Information) and revealed the characteristic of a strong liquid that vitrifies to a brittle glass. Furthermore, nanoindentation was applied to measure the mechanical property of the ZIF‐8 and GZIF*_m40_* membranes with substrates (Figure 2d; Figure S22, Supporting Information). Compared with ZIF‐8, the GZIF*_m40_* membrane had greater Young's modulus of 17.5 GPa, verifying the existence of glass phase. Relative to other MOF glasses and MOF crystal‐glass composites, the Young's modulus of GZIF*_m40_* was reasonable and relatively superior (Table S1, Supporting Information). This superior rigidity of GZIF*_m40_* was contributed by the factors of enhanced Zn‐ClBIM bond energy and reduced bond angle,^[^ ^10l^ ^]^ nonbonded interactions formed by ClBIM groups,^[^ ^10e,g^ ^]^ possible high density,^[^ ^10a^ ^]^ and size effect,^[^ ^10^, ^17^ ^]^ as discussed in Supporting Information (Figure S22, Supporting Information).

Synchrotron x‐ray total scattering data of ZIF‐8 and GZIF*_m40_* were collected to calculate the simplified structure factor *S(Q)* and Fourier transformed to obtain pair distribution function (PDF) in the form of *G(r)* (Figure 2e; Figure S23, Supporting Information). Although the diffraction peaks of GZIF*_m40_* were sharply weakened compared with those of ZIF‐8, there was a small amount of crystal phase in GZIF*_m40_*, which agreed with the XRD results. Peak position of PDF reflects interatomic distance. It was clear that ZIF‐8 had short‐/long‐range ordering structures, while the glass phase in GZIF*_m40_* was short‐range ordered but long‐range disordered, despite the weak oscillations over 7.0 Å from the contained ZIF‐8 with periodic structure (Figure 2e). In the short‐range correlation of ZIF‐8, the peaks at 2.00, 2.88, and 3.74 Å represented the Zn‐N, Zn‐C, and Zn‐C_(‐CH3)_ distances, respectively. For GZIF*_m40_*, the interatomic distances of Zn‐N, Zn‐C, and Zn‐C_(‐CH3)_/Zn‐Cl slightly shifted to 1.96, 3.00, and 3.58 Å, respectively. These variations were in line with the FTIR blue‐shift and interpreted by the differences of metal‐linker bond properties and functional groups. Both ZIF‐8 and GZIF*_m40_* had the Zn‐Zn interatomic distance peak of metal‐linker‐metal. These data proved the metal‐linker connections of ZIF‐8 and GZIF*_m_* and the changed bond environments of GZIF*_m_*. We simulated the Zn‐MeIM, Zn‐ClBIM, and MeIM‐Zn‐ClBIM connections by using density functional theory (Figure 2f; Figure S24, Supporting Information). Since the polar chlorine affects the chemical environment and electron cloud (as identified by Zn 2p XPS), the Zn‐ClBIM‐Zn had smaller angle and greater bond energy than Zn‐MeIM‐Zn. Therefore, the ClBIM in GZIF*_m_* could not be regularly arranged in the frameworks for crystallization, thereby disrupting long‐range MOF periodicity and prompting glass phase formation. These discrepancies in bond angles and strengths also contributed to the differences in atom‐atom distances of ZIF‐8 and GZIF*_m40_*.

### 2.4 Porous and Adsorptive Features

**Figure 3:**
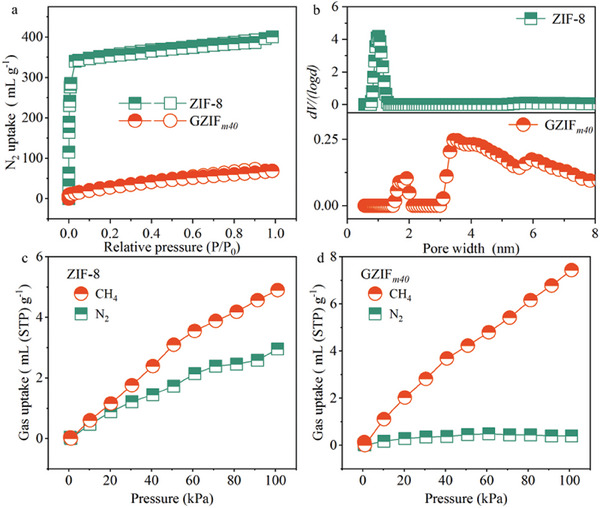
Porous and sorption properties. a) N_2_ adsorption‐desorption isotherms of ZIF‐8 and GZIF*_m40_* at 77 K. b) Pore width distributions of ZIF‐8 and GZIF*_m40_* calculated by quenched solid density functional theory with slit/cylindr.‐pore model based on N_2_ isotherms. c) CH_4_ and N_2_ sorption isotherms of ZIF‐8 at 25 °C. d) CH_4_ and N_2_ isotherms of GZIF*_m40_* at 25 °C.

We collected the N_2_ isotherms of ZIF‐8 and GZIF*_m_* at 77 K (**Figure** **3**a; Figure S25, Supporting Information). ZIF‐8 showed type‐I isotherms of microporous materials and had Brunauer‐Emmett‐Teller specific surface area of 1495 m^2^ g^−1^ (Table S2, Supporting Information). Comparatively, the GZIF*_m_* powders had poorer porosity, with specific surface areas of 865 m^2^ g^−1^ for GZIF*_m10_* and 115 m^2^ g^−1^ for GZIF*_m40_*. This porosity deterioration of GZIF*_m_* calculated by N_2_ isotherms was consistent with that of the MOF glasses from melting‐quenching,^[^ ^11d^ ^]^ because of the diffusion limitation of N_2_ into the narrowed pores at cryogenic temperature.^[^ ^11^, ^18^ ^]^ Pore width distributions calculated by various methods indicated the existed cavities in ZIF‐8 (Figure 3b; Figure S26, Supporting Information). Because of the glass ultramiroporous property, the pore peak intensity of GZIF*_m_* was attenuated (Figure 3b; Figures S27, S28, Supporting Information).

Adsorption isotherms for gases were collected at 25 °C (Figure 3c,d; Figure S29, Supporting Information). All samples showed sorption capacity in the order of CO_2_, CH_4_, and N_2_, due to the differences in gas condensability. Distinct from the porosity decline and the reduction of N_2_ uptake from framework collapse,^[^ ^11a^ ^]^ the CO_2_ and CH_4_ uptakes of GZIF*_m_* were even higher than those of ZIF‐8 owing to the more adsorption sites in glass phase. This phenomenon has also been found in previous studies,^[^ ^10^, ^11^, ^18^, ^19^ ^]^ in which the MOF glasses had low N_2_ uptake at 77 K but could adsorb CO_2_ and CH_4_ due to framework contraction but still remained microporosity. As calculated by ideal adsorption solution theory (IAST), the selectivity of CH_4_/N_2_ mixture (50:50) raised from 1.7 for ZIF‐8 to 10.3 for GZIF*_m40_* at 100 kPa, derived from the amelioration of CH_4_ sorption but deterioration in N_2_ uptake (Figures S30 and S31, Supporting Information). This result indicated the preferential sorption of GZIF*_m_* for CH_4_. It should be noted that the gas separation of CH_4_ and N_2_ is critical to natural gas and biogas purifications but is extremely difficult due to similar properties.

Density functional theory was utilized to simulate the interactions between functional groups and gas molecules (Figure S32, Supporting Information). Because linker‐mixed GZIF*_m_* was directly synthesized by gel‐thermal processing rather than melting‐quenching of MOFs with defined structures, the accurate model of glasses was difficult to determine, thus the six‐membered window from ZIF‐8 was used for simulation. For methyl in ZIF‐8, the binding energies of CH_4_ and N_2_ were ‐0.73 and ‐0.19 kJ mol^−1^, respectively. After replacing MeIM with ClBIM, the chlorine and phenyl groups in GZIF*_m_* had much stronger van der Waals interactions with CH_4_ than that in ZIF‐8, with binding energies of −2.72 and −2.14 kJ mol^−1^, respectively. However, the binding forces with N_2_ were slightly changed to −0.61 and −0.09 kJ mol^−1^, respectively (Figure S32, Supporting Information). These simulations could provide circumstantial evidence that chlorine and phenyl of ClBIM reinforced the preferential CH_4_ affinity and GZIF*_m_* had better binding capability and more affinity sites than ZIF‐8, although the binding energies of ZIF*_m_* were calculated based on an unrealistic structure. This consequently improved the selectivity and kept adsorption capacity of GZIF*_m_* for CH_4_. As reported in literature,^[^ ^11c^ ^]^ the abundant ultramicropores and unsaturated metal centers in glasses would considerably contribute to selective and preferential adsorption.

### 2.5 Preparation and Performance of GZIF*_m_* Membranes

**Figure 4:**
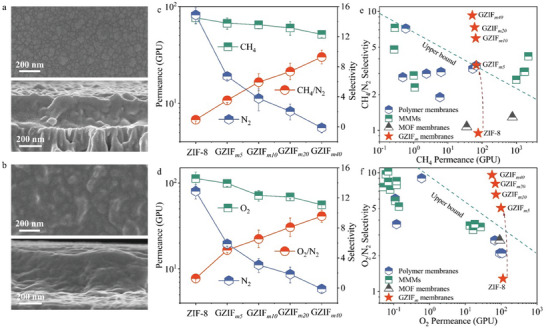
Preparation and separation performance of ZIF‐8 and GZIF*_m40_* membranes. a) Top and cross‐sectional view SEM images of a ZIF‐8 membrane. b) Top and cross‐sectional view SEM images of a GZIF*_m40_* membrane. c) Gas permeance and selectivity of the GZIF*_m_* membranes with different linker ratios for the CH_4_/N_2_ mixture. d) Gas permeance and selectivity of the GZIF*_m_* membranes with different linker ratios for the O_2_/N_2_ mixture. e) Comparison of the CH_4_/N_2_ separation performance of the ZIF‐8 and GZIF*_m_* membranes with polymer membranes, MOF membranes, and mixed‐matrix membranes (MMMs) (Table S3, Supporting Information). An upper bound was plotted from the advanced membranes. f) Comparison of the O_2_/N_2_ separation performance of the ZIF‐8 and GZIF*_m_* membranes with polymer membranes, MOF membranes, and MMMs (Table S4, Supporting Information). The upper bound was converted from the 2008 Robeson's plot^[^ ^3b^ ^]^ by using a membrane thickness of 10 µm.

We processed the glass composites on porous substrates to fabricate membranes (Figure S33, Supporting Information). Scanning electron microscopy (SEM) images indicated that the ZIF‐8 membrane was polycrystalline and continuous (**Figure** **4**a; Figures S34, S35, Supporting Information). Interestingly, the GZIF*_m_* membranes consisted of continuous glass matrices with dispersed ZIF‐8 nanocrystals (Figure 4b; Figures S36–S39, Supporting Information). In other words, the possible defects that usually existed in MOF membranes were eliminated by glass phase for the GZIF*_m_* membranes. Cross‐sectional SEM images showed the GZIF*_m_* membranes were slightly thicker than ZIF‐8, which might be interpreted by the more loading of precursors. Even though, the GZIF*_m40_* membrane was still much thinner than the melt‐quenched MOF glass membranes with thickness of tens to hundreds of micrometers. Small portions of ZIF‐8 and GZIF*_m_* injected into substrates would be beneficial to the membrane stability and compatibility. Compared with conventional synthesis of MOF membranes that require dozens of milliliters of solutions, the solvent and precursor consumptions of our method were reduced by two orders of magnitude, and the procedures analogous to those for polymer‐based membranes were more straightforward,^[^ ^20^ ^]^ subsequently reducing fabrication cost and improving environmental friendliness and scalability. Furthermore, the conditions of linker‐mixing gel‐thermal processing (i.e., 120 °C and normal room atmosphere) were milder and simpler than those of melt‐quenched method, thereby facilitating processability and diversifiability, such as fabrication of the MOF crystal‐glass composite membranes with polymer hollow fiber substrates (Figure S40, Supporting Information).

Gas permeation properties of the membranes were evaluated at 25 °C and 100 kPa. The GZIF*_m40_* membrane exhibited permeances ranged by H_2_, CO_2_, O_2_, CH_4_, and N_2_ (Figure S41, Supporting Information). It should be noted that the GZIF*_m40_* membranes had CH_4_ permselectivity (8.1) over N_2_ (Figure S42, Supporting Information). From the perspective of sorption/solution‐diffusion mechanism,^[^ ^21^ ^]^ which has usually been used to discuss the mechanism of polymer membranes and has also been extensively employed to discuss the separation of MOF membranes,^[^ ^22^ ^]^ the sorption‐diffusion coefficients and selectivites could be calculated based on isotherms and permeances. Although the porosity deterioration of GZIF*_m40_* was unfavorable to gas uptakes, the strong affinity of phenyls, halogens, and unsaturated metal centers to CH_4_ could offset this deterioration. Thus, the CH_4_ sorption coefficient increased by 52%, from 7.35 (ZIF‐8) to 11.2 (GZIF*_m40_*) × 10^−2^ cm^3^ cm^−3^ cmHg^−1^, while the N_2_ sorption coefficient decreased by 87%, from 4.43 to 0.59 × 10^−2^ cm^3^ cm^−3^ cmHg^−1^. Correspondingly, the CH_4_/N_2_ sorption selectivity of 18.8 for GZIF*_m40_* was ≈11 times as that for ZIF‐8 (1.66). Because of the narrowed channels of amorphous phase, both CH_4_ and N_2_ diffusion coefficients became inferior, from 2.97 and 5.37 × 10^−8^ cm^2^ s^−1^ for ZIF‐8 to 1.54 and 3.57 × 10^−8^ cm^2^ s^−1^, accompanied by CH_4_/N_2_ diffusion selectivity of 0.55 for ZIF‐8 and 0.43 for GZIF*_m_*. In terms of the sorption‐diffusion differences between ZIF‐8 and GZIF*_m_*, the selectivity improvement was mainly contributed by the adjustment of adsorption. In other words, although CH_4_ diffused slower in membranes than N_2_ and the CH_4_/N_2_ diffusion selectivity of GZIF*_m40_* was slightly poorer than that of ZIF‐8, the greatly improved sorption for CH_4_ with larger condensability and the strictly suppressed N_2_ sorption would boost the actual partial pressure, concentration gradient, and driving force for mass transfer, consequently resulting in preferential sorption separation. Certainly, the continuity and defect‐free property of the membranes were the prerequisites for gas separation.

We further investigated the separation performance of the GZIF*_m_* membranes with different ClBIM contents for CH_4_/N_2_ mixture (Figure 4c). As expected, the mixture selectivity and permeance of GZIF*_m_* exhibited upward and downward trends as the ClBIM increased, respectively. This was because more glasses provided stronger affinity to CH_4_, more ultramicropores, and better defect‐free property. For GZIF*_m40_*, the mixture CH_4_/N_2_ selectivity reached at 9.3, accompanied by a permeance of 51.6 GPU. This selectivity was better than that measured by single gas permeation (8.1), which was attributed to the competition of two gases for transport channels and the preferential CH_4_ sorption.^[^ ^22^, ^23^ ^]^ We compared the performance of GZIF*_m40_* with other highly efficient membranes (Figure 4e; Table S3, Supporting Information). For CH_4_/N_2_, MOF membranes usually showed almost no permselectivity, polymer membranes had selectivity ≈3.0, and mixed‐matrix membranes (MMMs) gave selectivity below 7.0. Our GZIF*_m40_* membrane exhibited approximately triple selectivity as polymer membranes. A polymer membrane and a MMM had selectivity over 7.0, but the CH_4_ permeance was less than 0.6 GPU. This permeance value was ≈2 orders of magnitude smaller than that of GZIF*_m40_*. Even relative to the most advanced ZIF‐62 glass foam membrane,^[^ ^11c^ ^]^ the GZIF*_m40_* membrane displayed lower permeance but 1.5 times selectivity. Meanwhile, the processability and processing conditions, e.g., no need for argon protection, high‐pressure treatment, grinding, and high‐temperature melting, constitute practical advantages.

Considering the greater van der Waals interactions of phenyls, halogens, and metal centers with O_2_ than N_2_ as shown in simulations and previous studies (Figure S32, Supporting Information),^[^ ^11^, ^14^, ^24^ ^]^ as well as the smaller kinetic diameter of O_2_ than that of N_2_, the single‐gas permeation property and mixture separation performance of GZIF*_m_* for O_2_/N_2_ were also assessed (Figure 4d; Figure S43, Supporting Information). Gas separation for O_2_/N_2_ plays an important role in chemical and medical industries, but their similarities hamper separation. Similar to the performance for CH_4_/N_2_ separation, more glasses in GZIF*_m_* led to higher selectivity but lower permeance. The GZIF*_m40_* membrane showed much better performance than ZIF‐8, with selectivity up to 9.6 and permeance of 55.6 GPU. For the O_2_/N_2_ system, the GZIF*_m40_* membrane outperformed most polymer, MOF, and MMMs (Figure 4f; Table S4, Supporting Information).

## 3 Conclusion

We have developed a linker‐mixing concept based on gel‐thermal processing to directly fabricate thin linker‐mixed MOF crystal‐glass composite membranes, through a combination of gel‐coating and thermal treatment. Mixed linkers with different bond strengths and angles to metal centers facilitate the formation of glass phase, avoiding generation of crystalline defects which usually exist in MOF membranes. Meanwhile, our GZIF*_m_* membranes are fabricated without the need for massive solvents and precursors, high temperature and pressure, protection by vacuum or inert gas, and complex regulation of precursor diffusion and crystallization, which are usually critical to the synthesis of MOF‐crystalline and MOF glass membranes. These superiorities allow the membranes to be processed in a simple, controllable, scalable, cost‐effective, and environmentally friendly route. In addition, because the halogens and phenyls, unsaturated metal centers, and ultramicropores of glasses promote specific sorption, the membranes display great performance for CH_4_/N_2_ and O_2_/N_2_ separations. Overall, the concepts of linker‐mixing mechanism and gel‐thermal processing for MOF crystal‐glass composite formation inspire the design of efficient membranes and materials for various applications.

## Conflict of Interest

The authors declare no conflict of interest.

## Supporting information



Supporting Information

## Data Availability

The data that support the findings of this study are available from the corresponding author upon reasonable request.
