# Supplementary material for: Directly Gel‐Thermal Processing of Linker‐Mixed Crystal‐Glass Composite Membranes for Sorption‐Preferential Gas Separation
Source: Adv Sci (Weinh). 2024 Dec 12;12(5):2413942. doi: 10.1002/advs.202413942 (PMC11791987; doi:10.1002/advs.202413942)
Supplement: Supplementary file 1 — Supporting Information [file ADVS-12-2413942-s001.pdf]

ADVANCED SCIENCE
Open Access

Supporting Information

for *Adv. Sci.*, DOI 10.1002/advs.202413942

Directly Gel-Thermal Processing of Linker-Mixed Crystal-Glass Composite Membranes for Sorption-Preferential Gas Separation

Yihao Xiao, Yanqing Yu, Xinxi Huang, Da Chen and Wanbin Li**

((Supporting Information can be included here using this template))

Supporting Information

Directly Gel-Thermal Processing of Linker-Mixed Crystal-Glass Composite Membranes for Sorption-Preferential Gas Separation

Yihao Xiao[#], Yanqing Yu[#], Xinxi Huang, Da Chen, and Wanbin Li**

((Please insert your Supporting Information text/figures here. Please note: Supporting Display items, should be referred to as Figure S1, Equation S2, etc., in the main text…)

Materials and Methods

Materials

Anodic aluminum oxide (AAO) substrate with pore size of 20 nm was purchased from GE Healthcare Whatman. Zinc acetate dihydrate ($Zn(CH_3COO)_2 \cdot 2H_2O$, 98%), 2-methylimidazole (HMeIM, 98%), 2-chlorobenzimidazole (HClBIM, ≥ 95%), and ethanolamine (99%) were purchased from Shanghai Macklin Biochemical Technology Co., LTD, China. Zinc nitrate hexahydrate ($Zn(NO_3)_2$ $6H_2O$, ≥ 99%) was purchased from Guangzhou chemical reagent factory, China. Ethanol (≥ 99.7%) and methanol (≥ 99.5%) were purchased from Guangdong Guanghua Sci-Tech Co., China.

Fabrication of Gels

Zinc acetate dihydrate (3.47 g) was added in ethanol (10.0 mL) and stirred at 60 ^oC for 20 min to obtain a suspension. Ethanolamine (1.0 mL) was dropped into the suspension with stirring at 60 ^oC for 20 min to fabricate the transparent sol. To obtain precursor gel, the mixture of 2-methylimidazole (HMeIM) and 2-chlorobenzimidazole (HClBIM) with different molar ratios was dispersed into the prepared sol with stirring. Wherein, the metal/linker molar ratio was 1:4; and the molar ratio of HClBIM in linker mixture was varied from 5% to 60%. After vigorous stirring, the viscous gel was generated due to the formations of the ZIF nanocrystals and zinc-based colloids and complexes.

Processing of ZIF-8 and GZIF$_m$

For synthesis of ZIF-8 or GZIF$_m$, the gel (1.0 mL) was diluted by solvent (7.0 mL) and spin-coated on a substrate with rotational speed of 8000 revolutions per minute. The dip-coating method was used to prepared samples on polyvinylidene fluoride hollow fiber, the substrates were immersed into the gel for 30 s. Then, the coated layer and substrate were thermally treated at 120 ^oC in a normal room atmosphere for 40 h to volatilize solvent molecules,

additives, and excessive linkers. Under the thermal condition, the zinc-linker coordination reactions were carried out for transforming gel layer to gel film. For the convenience of characterizations, the ZIF-8 or GZIF$_m$ film was scraped from the substrate to obtain the powder sample. In order to observe the internal structure by transmission electron microscopy, the gel with different linker ratios was spin-coated and thermally transformed to glass on the carbon film with copper mesh support.

Synthesis of ZIF-8 by Solvothermal Method

$Zn(NO_3)_2$ $6H_2O$ (0.30 g) and HMelM (0.66 g) were separately dissolved in methanol (14.0 mL). These two solutions were mixed and transferred into a Teflon lined stainless steel autoclave, and then treated at 150 ^oC for 12 h. After reaction, the ZIF-8 powder was washed with methanol for three times and collected by centrifugation at 5000 rpm for 6 min. Finally, the powder was dried at 80 ^oC under vacuum for 12 h.

Gas Permeation Property

Gas permeation property was measured by using a reequipped gas permeation analyzer (MGT-01, Labthink Co.). Gas permeation was evaluated at temperature of 25 ^oC and transmembrane pressure of 100 kPa. For single gas permeation, the membrane with effective area of 3.14 cm^2 was sealed in cell by using O-ring. To remove any possible impurities, the permeation system was vacuum-treated for two hours. Then, the gas was injected into the feed side of the cell, and the permeate side was vacuum-treated for gas permeation. After a period of permeation, the permeate gas volume was measure by using a gas chromatograph (GC-6890 with columns of TDX-1 and molecular sieve 5A). Gas permeation was tested in the order of H_2, CO_2, O_2, N_2, and CH_4. Gas permeance (P, 1 GPU = 10^{-6} cm^3 (STP)) cm^{-2} s^{-1} $cmHg^{-1}$) and permeation selectivity (α) were calculated as the equations of (1) and (2), respectively.

$$P = \frac{1}{\Delta p} \times \frac{1}{A} \times \frac{\Delta V}{R \times T \times \Delta t}$$

(1)

$$\alpha = \frac{P_i}{P_j}$$

(2)

Where P was gas permeance; Δp, ΔV, and Δt was transmembrane pressure, permeate volume, and permeation time, respectively; A was effective membrane area; R was gas constant value; T was temperature; P_i and P_j were permeances of gas i and j, respectively.

Separation Performance

Gas separation performance was evaluated by using a self-made apparatus. Gas separation was performed at temperature of 25 ^{o}C in room atmosphere. For gas separation, the membrane with effective area of 1.76 cm^2 was sealed in permeation cell by using O-ring. Then, the gas mixture was injected into the feed side of the cell with a flow rate of 20 mL min^{-1} and the permeate side of the cell was swept by using argon with a flow rate of 10 mL min^{-1}. Gas composition and flow rate were controlled by using mass flow meter. After running steadily, a gas chromatograph (GC-9280 with columns of TDX-1 and molecular sieve 5A) was used to monitor the composition of the permeate gas mixtures. Gas permeance (P) and mixture selectivity (α) were calculated as the equations of (3) and (4), respectively.

$$P_i = \frac{V_m \times y_i}{A \times \Delta p}$$

(3)

$$\alpha = \frac{y_i / y_j}{x_i / x_j}$$

(4)

Where V_m was molar flow rate of permeate gases; A was effective membrane area; Δp was partial transmembrane pressure; y_i and y_i were the proportions of gas i and gas j at permeate side, respectively; x_i and x_j were the proportions of gas i and gas j at feed side, respectively.

Characterizations

Transmission electron microscopy (TEM) images were captured by using an electron microscope (JEM-2100, JEOL Ltd.) with an accelerating voltage of 200 kV. For fabrication of the ZIF-8 and $GZIF_m$ film samples, the related gels were coated and thermally treated on the carbon films with copper mesh supports. An attached x-ray energy dispersion spectroscope (EDX) was used to obtain the mapping images of element distribution.

Fourier transform infrared (FTIR) spectrophotometer (IRTracer-100, Shimadzu Co.) was employed to study the chemical structure of ZIF-8 and $GZIF_m$.

Proton nuclear magnetic resonance (^1H NMR) characterization was carried out by using an NMR apparatus (Ascend 600 MHz, Bruker Co.). Before characterization, the sample was digested by using trifluoroacetic acid-d. Peak areas were measured by using MestReNova software package.

X-ray diffraction (XRD) patterns were recorded on the x-ray diffractometers (D/Max 2500, Rigaku Co. and D2 PHASER, Bruker Co.).

X-ray photoelectron spectroscopy (XPS) experiment was carried out on an RBD upgraded PHI-5000C ESCA system (PerkinElmer) with an incident radiation of monochromatic Mg Kαx-rays (hv = 1253.6 eV).

Thermogravimetric analysis (TGA) in air atmosphere was performed on a Netzsch instrument (TG 209 F3, Germany) with a heating rate of 10 ^oC min^{-1} in the range of 30–800 ^oC. Data were exported by NETZSCH Proteus software package. For confirming the stability of the samples in the DSC scans, the cyclic TGA data were also collected by using a thermal

analyzer (TGA/DSC 3+, Mettler Toledo, Switzerland) with heating rate of 10 ^{o}C min^{-1} and range of 30–500 ^{o}C in N_2 atmosphere for two cycles. Data were exported by STARe SW 15.00 software package.

Differential scanning calorimetry (DSC) curves with exothermic downward direction were collected by using a calorimeter (TA Q2000, DSC2500, America). For the five scan cycles, the sample was heated with a rate of 10 ^{o}C min^{-1} from 30 to 500 ^{o}C in the N_2 atmosphere to perform the first upscan; then the sample was cooled with a rate of 10 ^{o}C min^{-1} to 150°C; and followed by cycling at the same rate of heating and cooling. For investigating the fragility index of the GZIF$_{m40}$, DSC upscans were performed at different heating rates, which equal the prior cooling rates. The T_f measured with the heating rate of 10 K min^{-1} was defined as T_g. Fragility index was determined as the slope of log(1/q)-T_g/T_f. To determine the heat capacity (C_p) of GZIF$_{m40}$, both the blank and the sapphire were measured for comparison.

Synchrotron x-ray total scattering data of ZIF-8 and GZIF$_{m40}$ were collected with the PD beamline from Australian Synchrotron using a Mythen II detector over 124º 2θ. Samples were packed into 0.7 mm borosilicate capillaries and rotated during data collection. Wavelength, zero error, and instrument contribution to peak shape were refined using NIST LaB6 660b. The scattering data were used to calculate the simplified structure factor *S(Q)* and be Fourier transformed to obtain pair distribution function (PDF) in form of *G(r)*. Q was the scattering vector whose magnitude was $4\pi \sin \theta/\lambda$, where 2θ was the scattering angle and λ was the incident radiation wavelength, 0.5905 Å. The PDF data were converted by PDFgetX3 software package.

A field-emission scanning electron microscope (SEM, Ultra-55, Zeiss Co.) with accelerating voltage of 3 kV was used to observed the morphology of the prepared membranes. Before observation, the sample was coated with a thin layer of gold.

Nitrogen adsorption-desorption isotherms were collected by using a physisorption analyzer (Autosorb iQ Station 1, Quantachrome Co.) at 77 K. Brunauer-Emmett-Teller (BET)

specific surface area was calculated based on N_2 adsorption-desorption isotherms at 77 K (P/P_0 range: 0.001–0.3, constant C > 0). Pore size distribution was calculated by nonlocal density functional theory (NLDFT) and quenched solid density functional theory (QSDFT) based on different pore types, i.e., slit, cylindr., and slit/cylindr. pore.

Gas adsorption isotherms were collected by using the physisorption analyzer (Autosorb iQ Station 1, Quantachrome Co.) at 25 ^{o}C. Before gas adsorption and desorption, the sample was degassed under vacuum conditions at 120 ^{o}C for 16 h.

A nanoindentation instrument (NanoTest Vantage, Micro Materials Ltd., UK.) was used to study the mechanical property of membranes. Average Young's modulus was calculated from the load-displacement data of the membrane sample with 3 measurements at different locations (0.1 mN).

Young's modulus was obtained by the following equation:

$$E = \frac{(1 - v^2)}{\frac{1}{E_r} - \frac{(1 - v_i{}^2)}{E_i}} \tag{5}$$

Where E_r was reduced modulus; E_i = 1141 GPa and v_i = 0.07 were known constant for Berkovich diamond indenter tip; and Poisson's ratio (v) was taken as 0.2 according to literature.[1–3]

IAST-Predicted Selectivity

Ideal adsorption solution theory (IAST) was used to predict mixture adsorption behavior. In order to perform the integration, the single-component isotherms were fitted using the single-site Langmuir-Freundlich equation (6):

$$q = \frac{abp^c}{1 + bp^c} \tag{6}$$

Where q was the adsorbed amount per mass of adsorbent (mmol g^{-1}); p was the pressure of bulk gas at equilibrium with the adsorbed phase (kPa); a was the saturation capacity (mmol g^{-1}); b was the affinity coefficient (1/kPa); and $1/c$ was the deviation from an ideal homogeneous surface. The fitted parameters were employed to predict the multicomponent adsorption.

Selectivity $S_{A/B}$ of components A and B was calculated as the equation of (7):

$$S_{A/B} = \frac{q_A/p_A}{q_B/p_B} \tag{7}$$

Where q_A and q_B were the amounts of the adsorbed A and B, respectively; and p_A and p_B were the partial pressures of A and B in the mixture, respectively.

Bond Energy Calculation

All quantum chemical calculations, including geometry optimizations and frequency calculations, were performed at the B3LYP/6-31G* level with quantum chemical package Gaussian 09.[4]

Bond energy (ΔE, kJ mol^{-1}) was calculated as equation (8):

$$\Delta E = 2625.5 \times (H_1 + H_2 - H_{total}) \tag{8}$$

Where H_{total} was enthalpy of optimized system; and H_1 and H_2 were enthalpy of part 1 and 2, respectively.

Adsorption Energy Calculation

Adsorption energy was calculated based on density functional theory (DFT) and performed by using a Castep module.[5] A generalized gradient approximation method with Perdew-Burke-Ernzerhof function was employed to describe the interactions between valence electrons and ionic core.[6,7] The adopted energy cut-off for the plane-wave basis was set as 450 eV and the

threshold values of convergence criteria were specified as follows: 0.002 Å for maximum displacement, 0.05 eV Å^{-1} for the maximum force, 0.1 GPa for the maximum stress, 10^{-5} eV atom^{-1} for energy, and 2.0×10^{-6} eV per atom for self-consistent field tolerance.

Adsorption energy (ΔE, eV) was calculated as the equation of (9):

$$\Delta E = (E_{total} - E_1 - E_2) \tag{9}$$

Where E_{total} was the energy of the optimized system; E_1 was the energy of ZIF-8; and E_2 was the energy of an optimized O_2, N_2 and CH_4 within a 10 Å × 10 Å × 10 Å box. 1 Ha = 27.212 eV = 2625.5 kJ mol^{-1}.

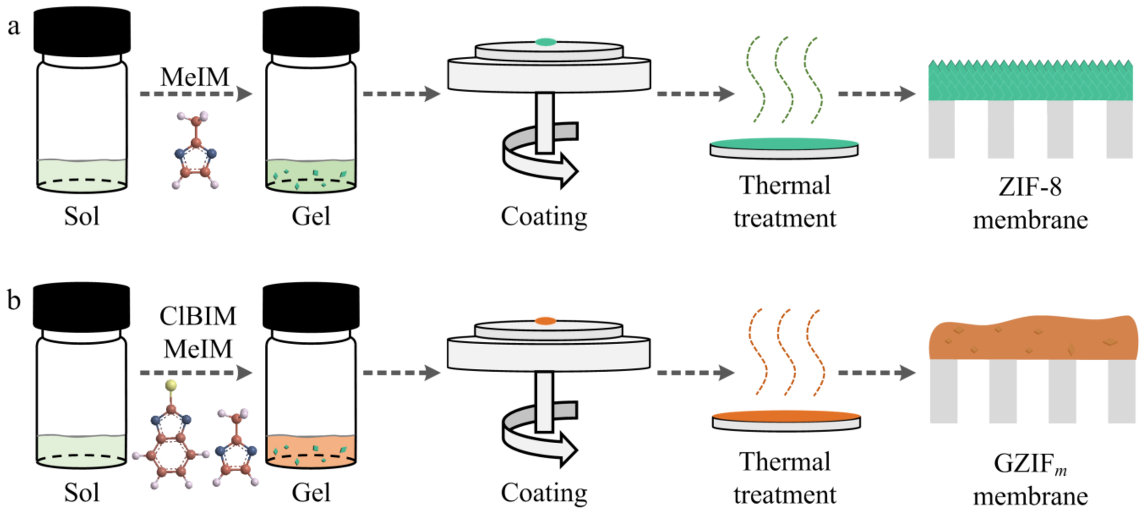

Figure S1. Gel-thermal processing procedures of (a) ZIF-8 and (b) GZIF$_m$.

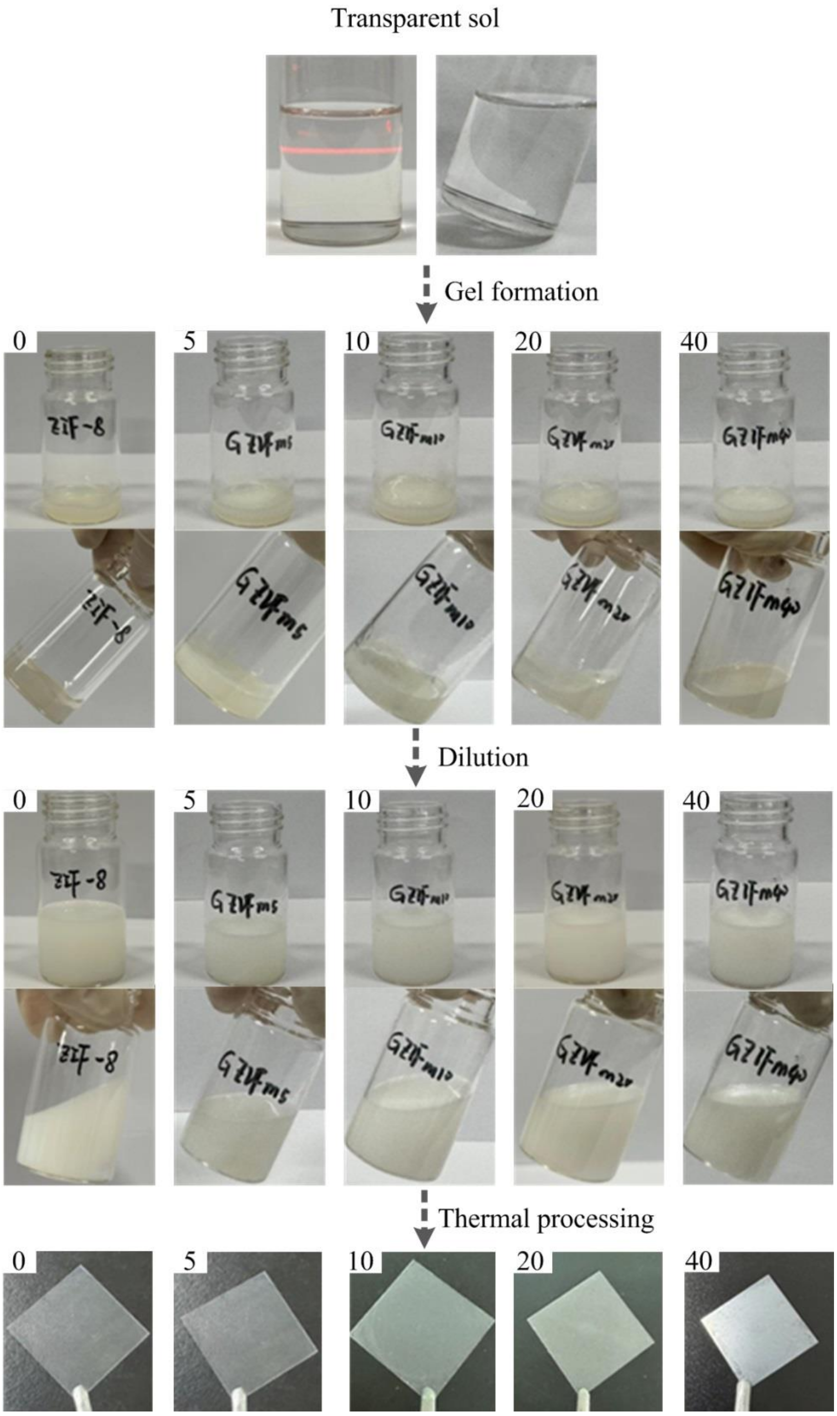

Figure S2. Digital photographs of the sol, the gels with precursor ClBIM molar ratios of 0%, 5%, 10%, 20%, and 40% before and after dilution, and the ZIF-8, GZIF$_{m5}$, GZIF$_{m10}$, GZIF$_{m20}$, and GZIF$_{m40}$ films on the cover glasses.

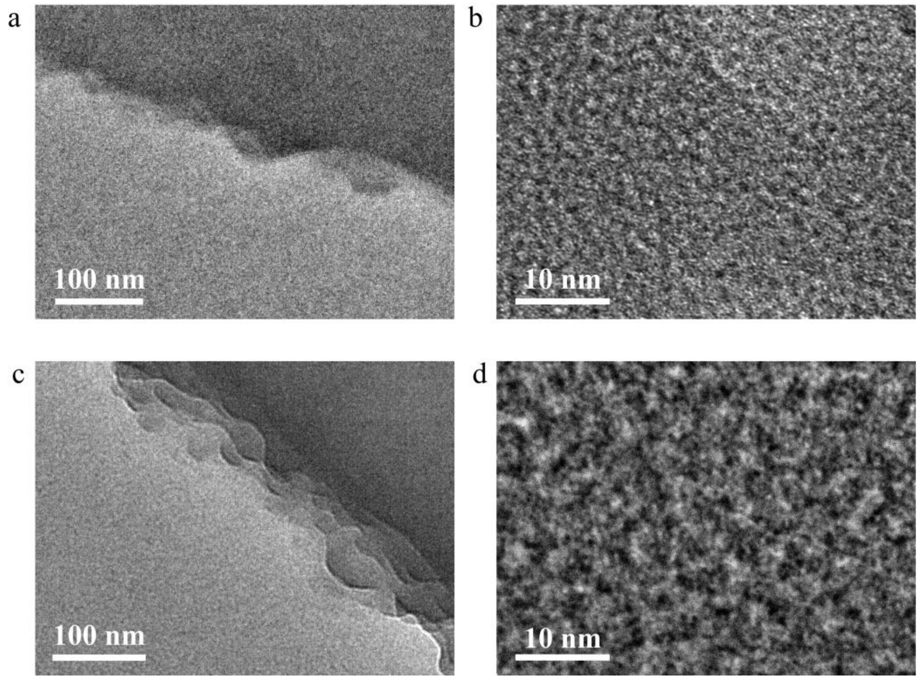

Figure S3. TEM images of (a,b) ZIF-8 and (c,d) GZIF$_{m40}$ with different magnifications.

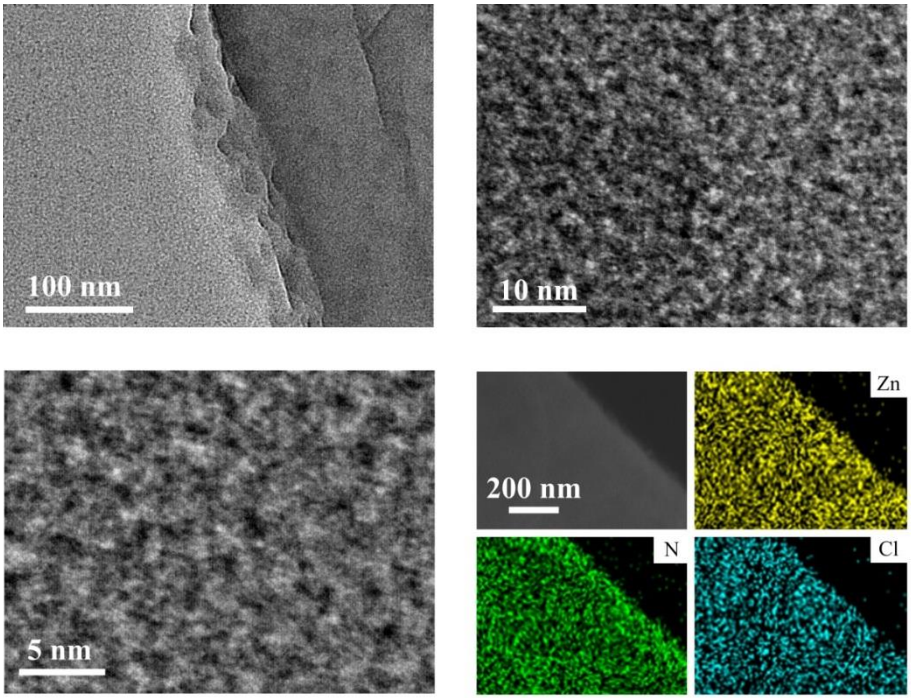

Figure S4. TEM and EDX mapping images of $GZIF_{m10}$.

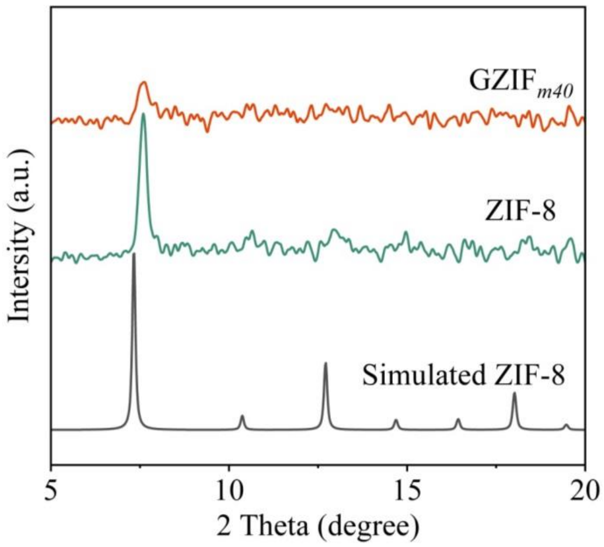

Figure S5. XRD patterns of ZIF-8 and GZIF$_{m40}$. The film samples were processed on the cover glass substrates. XRD pattern of simulated ZIF-8 is presented for comparison.

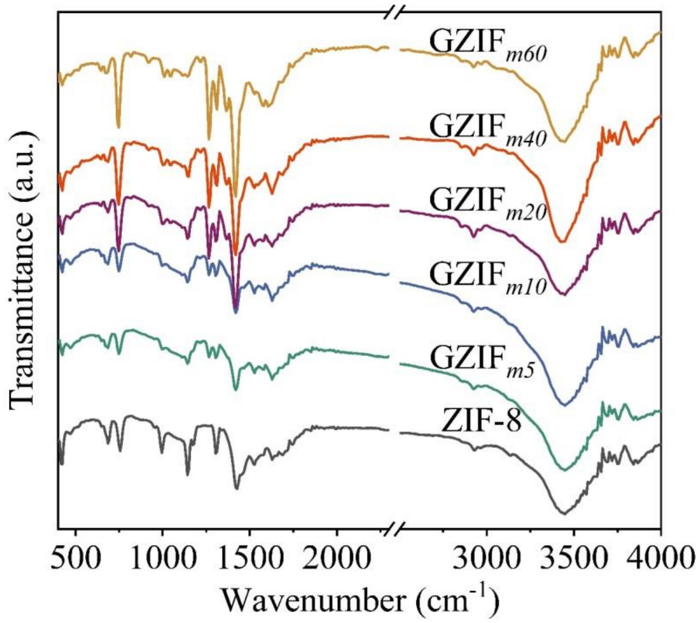

Figure S6. FTIR spectra of ZIF-8 and GZIF$_m$.

FTIR data manifested a typical ZIF-8 spectrum,[8–13] with peaks for Zn–N at 421 cm^{-1}, C–H at 754 cm^{-1}, and C–N at 996/1143 cm^{-1}. After the ClBIM addition, the peaks of ZIF-8 were retained, while the peaks for C–Cl at 816 cm^{-1} and phenyl C-H at 1267 cm^{-1} emerged in the FTIR spectra of GZIF$_m$ and were strengthened with the increment of ClBIM. A carboxylate peak from basic zinc acetate appeared at 1550 cm^{-1} in the FTIR spectra.

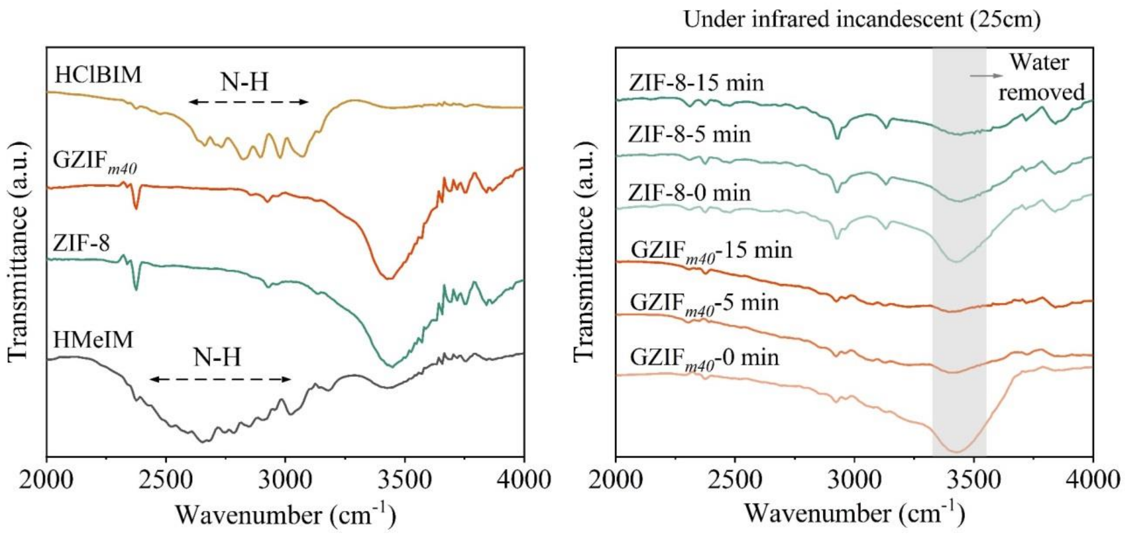

Figure S7. FTIR spectra of HMeIM, HClBIM, ZIF-8, and GZIF$_{m40}$.

Relative to the unreacted HMeIM and HClBIM linkers with protons and characteristic peaks of N–H (marked by dash arrows). Since the broad peak around 3500 cm^{-1} was related to water and N-H, the sample was characterized after drying under an infrared lamp. After irradiation for 15 min, the broad peak was sharply reduced, indicating that the signal should be attributed to -OH from water and basic zinc acetate. The absence of N–H indicated that the linkers in ZIF-8 and GZIF$_{m40}$ were deprotonated and coordinated with zinc centers.

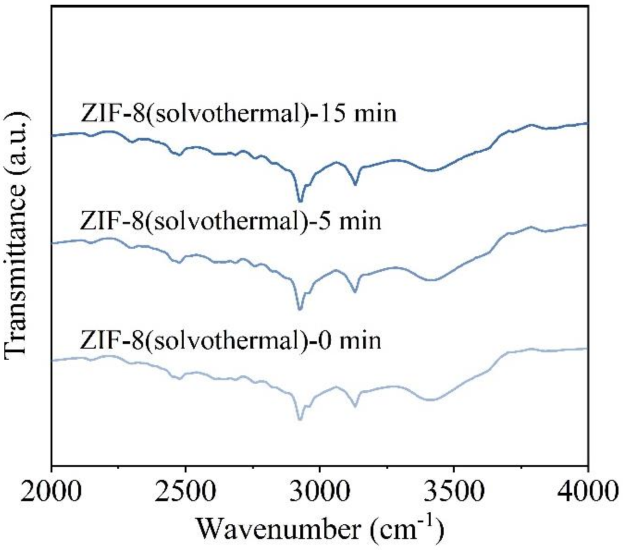

Figure S8. FTIR spectra of ZIF-8 prepared by solvothermal method.

The FTIR spectra of sample did not change obviously after drying under an infrared lamp.

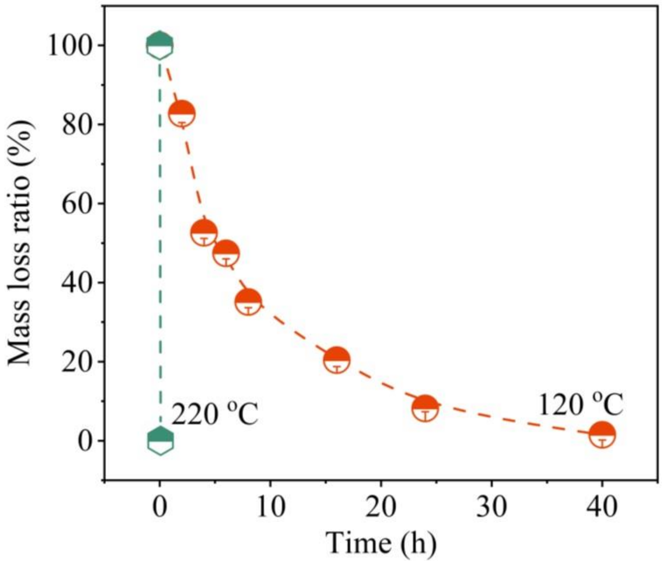

Figure S9. Mass loss ratios of HClBIM at 120 and 220 ^{o}C with the extension of time.

The temperature of 220 ^{o}C could make the HClBIM completely volatilize within 5 min.

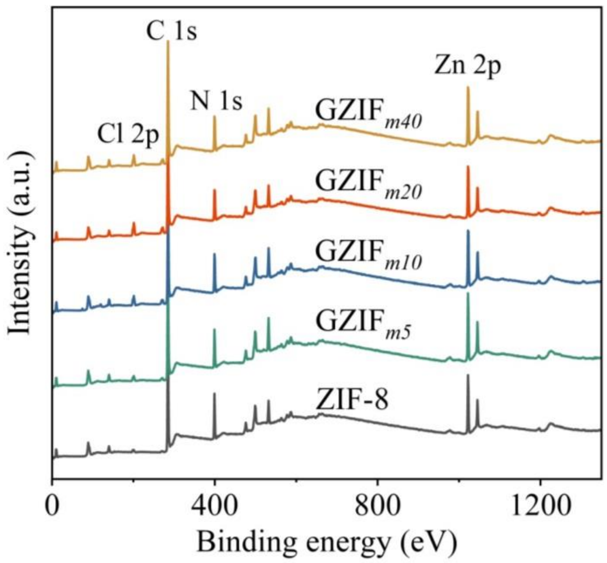

Figure S10. Full XPS survey spectra of ZIF-8 and GZIF$_m$.

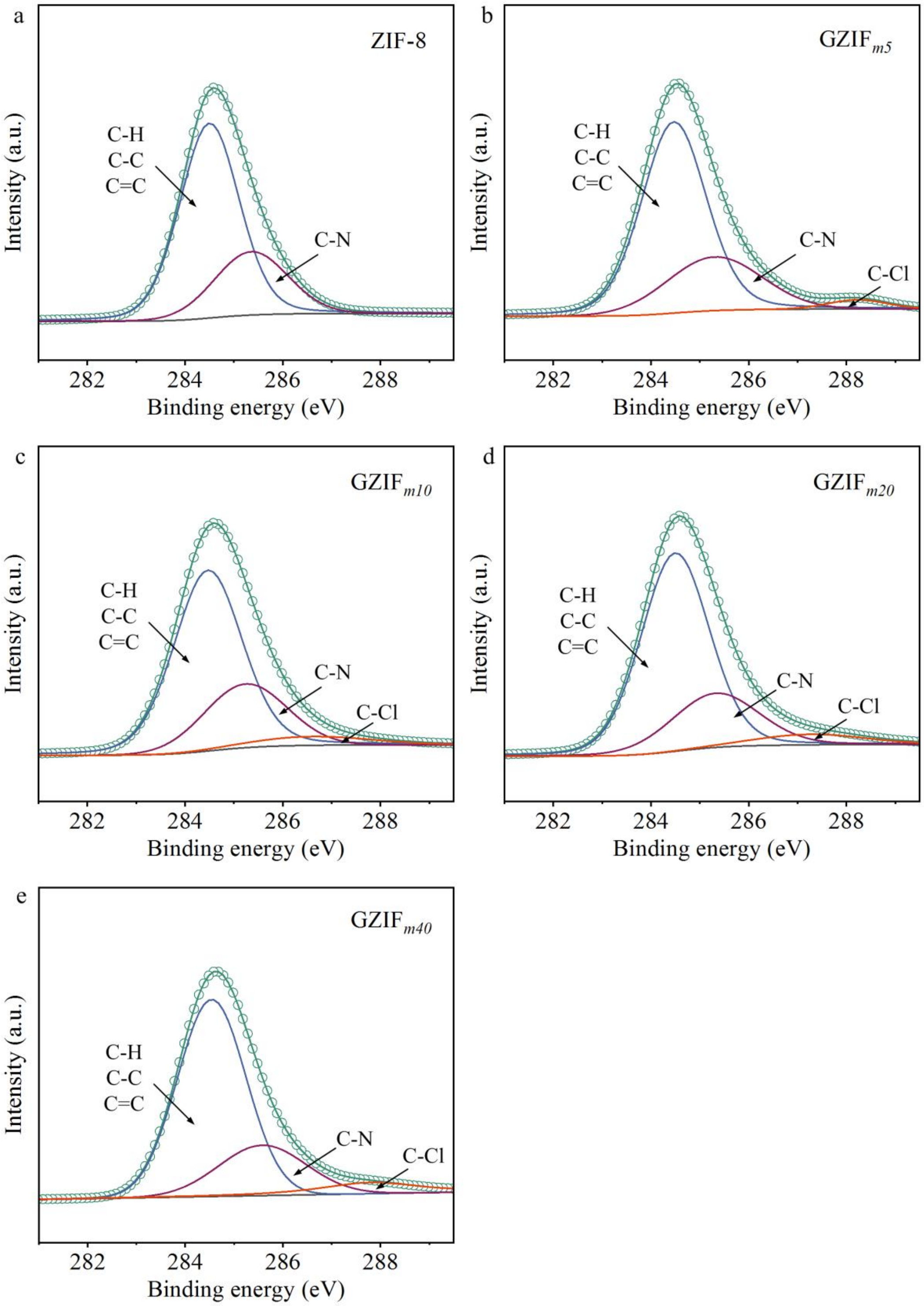

Figure S11. High-resolution C 1s XPS spectra of ZIF-8 and GZIF$_m$.

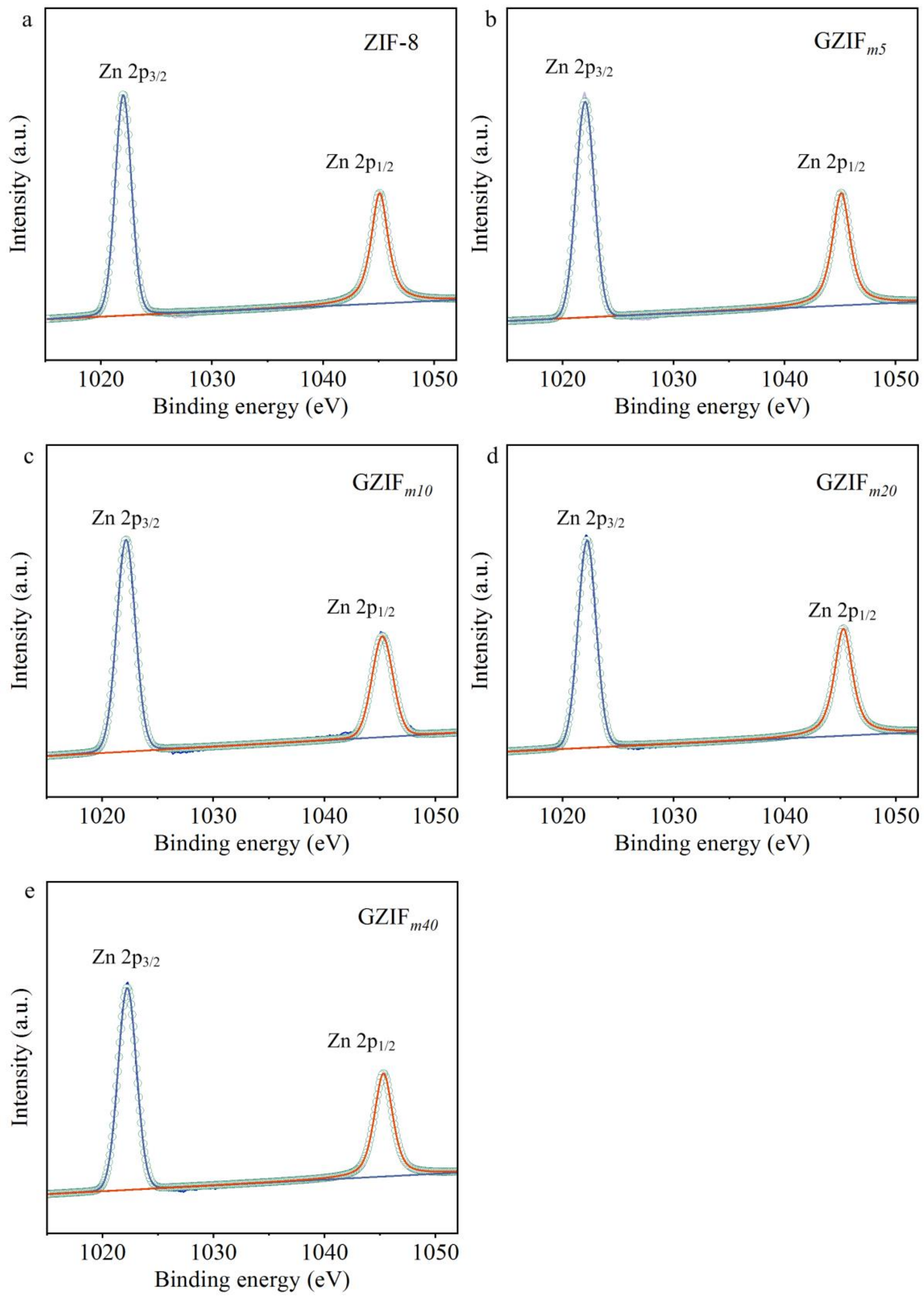

Figure S12. High-resolution Zn 2p XPS spectra of ZIF-8 and $GZIF_m$.

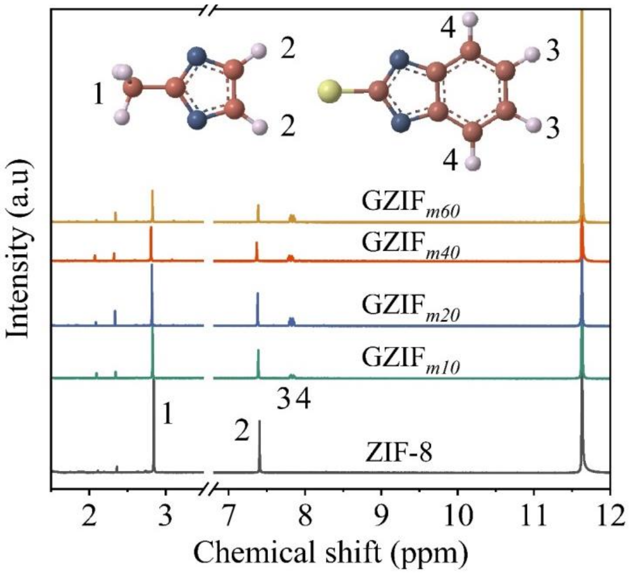

Figure S13. ^{1}H NMR spectra of ZIF-8 and GZIF$_m$. The peaks of hydrogen at different positions of MeIM and ClBIM were marked by 1, 2, 3, and 4.

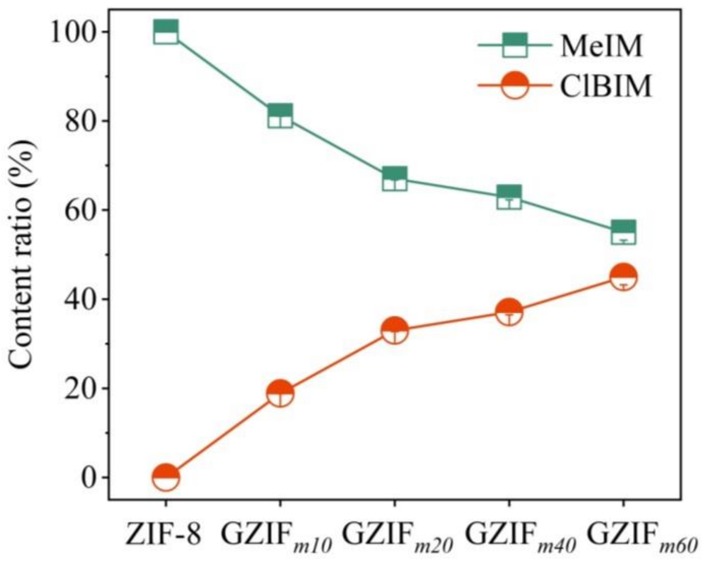

Figure S14. Linker ratios of GZIF$_m$. These values were calculated based on the peak areas of the ^{1}H NMR spectra.

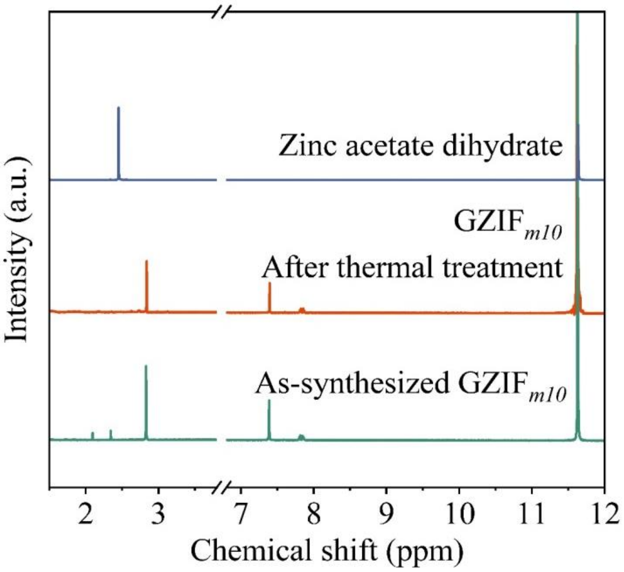

Figure S15. ^{1}H NMR spectra of zinc acetate dihydrate, as-synthesized GZIF$_{m10}$, and GZIF$_{m10}$ after thermal treatment at 220 ^{o}C for 3h.

In order to study whether there was unreacted linker, the GZIF$_{m10}$ sample was heated at 220 ^{o}C for 3 h. If the GZIF$_{m10}$ sample contained physically loaded linker molecules, they would be volatilized during heat treatment (Figure S9), thereby causing the change in linker ratio. Moreover, considering that HClBIM with higher melting point of 193.3 ^{o}C was more difficult to be volatilized than HMeIM (143 ^{o}C) and MeIM could be completely reacted, the ClBIM content should decrease after thermal treatment. However, the linker proportion of ClBIM in GZIF$_{m10}$ only slightly varied from 18.9% to 20.6% after thermal treatment. This result revealed that there was no physically loaded linker and all linkers were coordinated with metal centers. Notably, after thermal treatment, this acetate peak in the ^{1}H NMR was eliminated, proving the removal of basic zinc acetate.

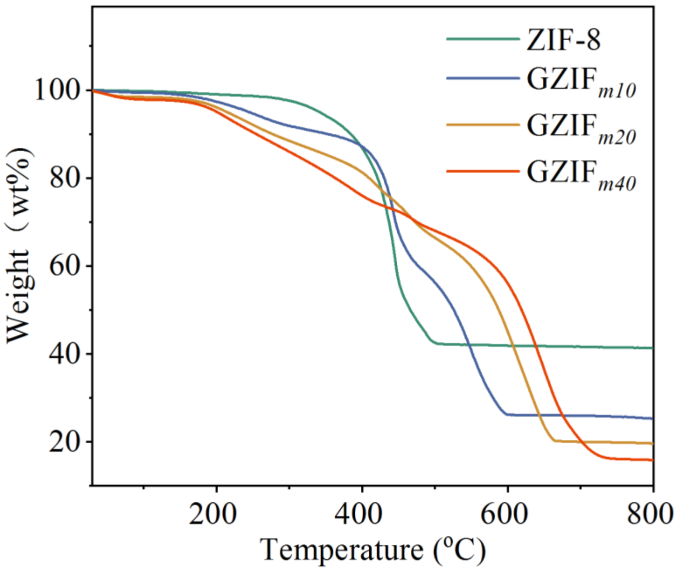

Figure S16. TGA profiles of ZIF-8 and GZIF$_m$ in air atmosphere.

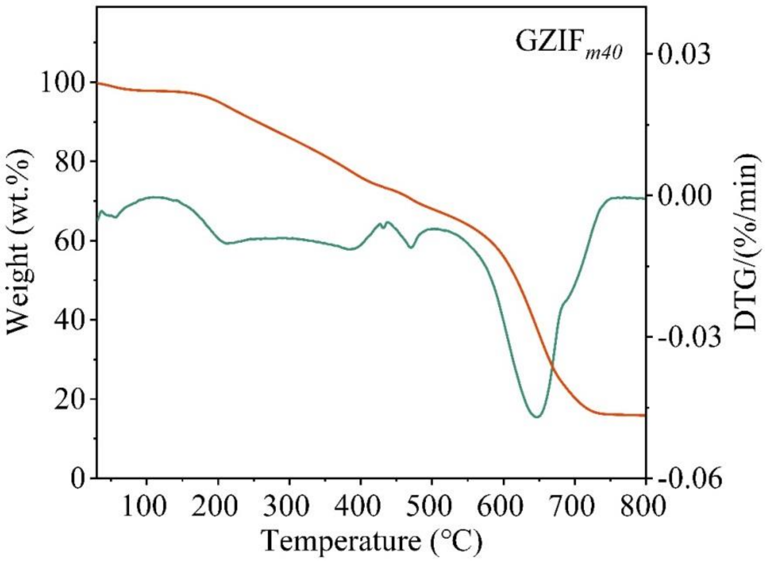

Figure S17. TGA and derivative TG (DTG) curves of GZIF$_{m40}$ in air atmosphere.

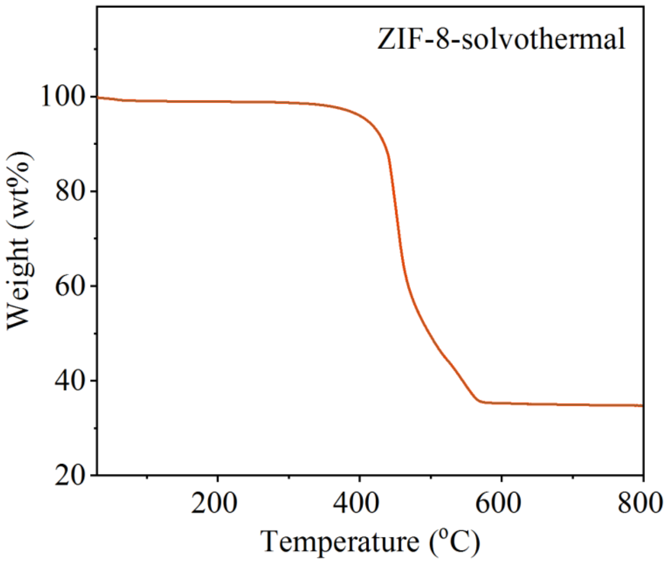

Figure S18. TGA profile of ZIF-8 synthesized by solvothermal method in air atmosphere.

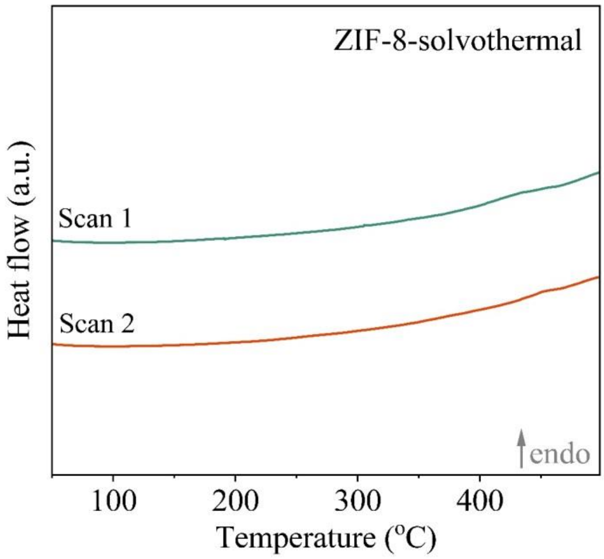

Figure S19. DSC upscans of ZIF-8 synthesized by solvothermal method.

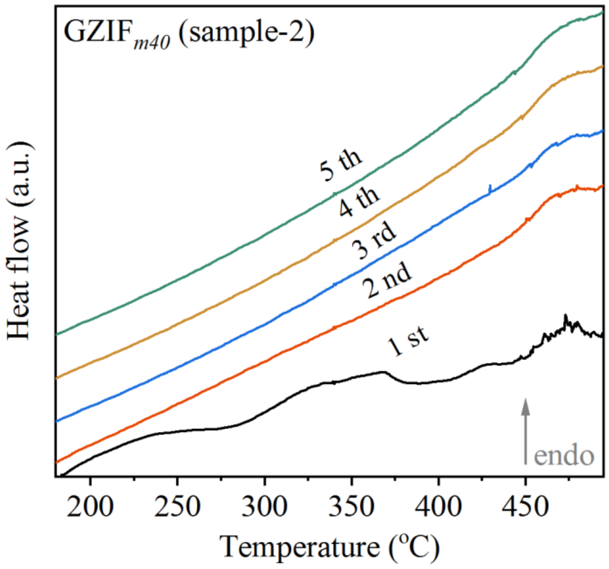

Figure S20. Cyclic DSC upscans of synthesized GZIF$_{m40}$ sample from different batches.

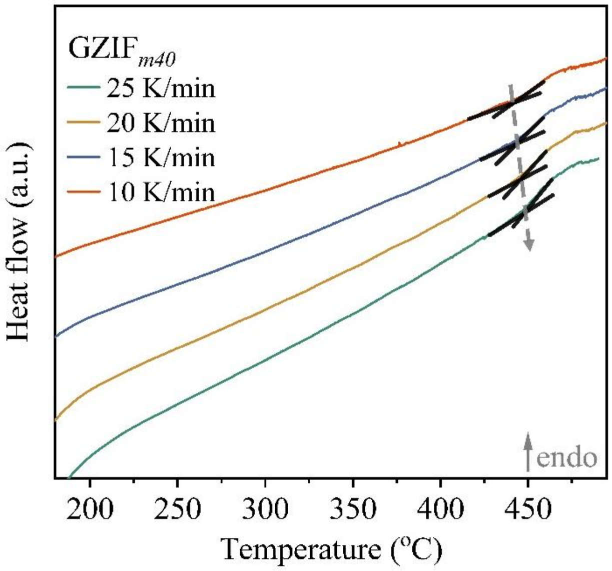

Figure S21. DSC upscans of GZIF$_{m40}$ measured at different heating rates. The heating rate was the same as prior cooling rate.

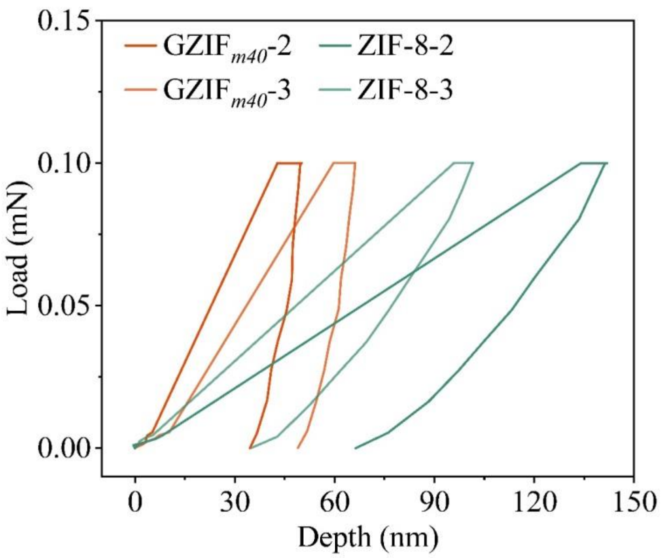

Figure S22. Load-displacement curves of ZIF-8 and GZIFm$_{40}$ obtained from nanoindentation at different locations.

The great Young's modulus value (17.5 ± 0.9 GPa) of GZIF$_{m40}$ could be attributed to the following four reasons. i, Coordination characteristics, the increased bond energy and decreased bond angle of GZIF$_{m}$, identified by DFT calculation, contributed to the enhanced rigidity. For example, the MOF glass of G-Mg-adp (adp, adipate) with highly deformed structure and strong coordination bonds showed large Young's modulus of 18.29 GPa.[14] ii, Nonbonded interactions, the ClBIM linker with chlorine and phenyl groups might introduce nonbonded interactions, thereby increasing Young's modulus.[15] For example, because the van der Waals forces provided strong intermolecular interactions in MOF glasses, the MOF glasses of Zn-DCI (DCI, 4,5-dicyanoimidazole) and Cd-DCI had Young's modulus as high as 126 and 24 GPa, respectively.[16] iii, Possible high density, large ClBIM increased the density and then stiffened materials.[1] iv, size effect, higher elastic modulus of materials and MOF glasses would be measured at smaller depths below 100 nm.[17,18] Since the GZIF$_{m40}$ membrane was thin, the indentation depth was small.

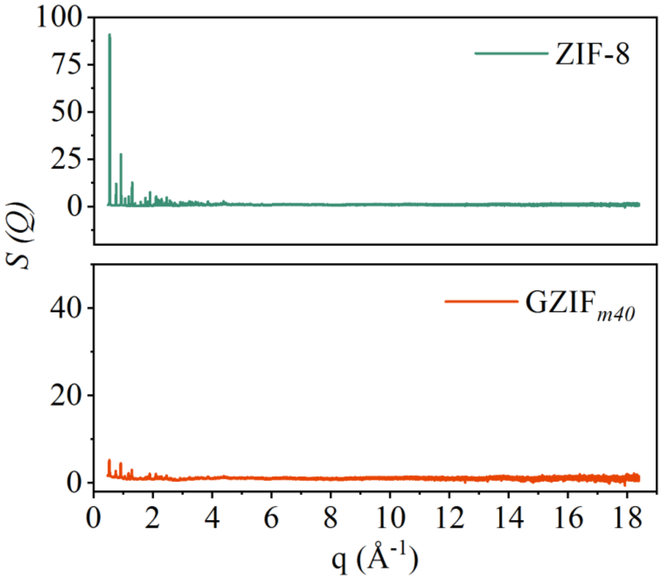

Figure S23. Synchrotron x-ray total scattering structure factors of ZIF-8 and GZIF$_{m40}$.

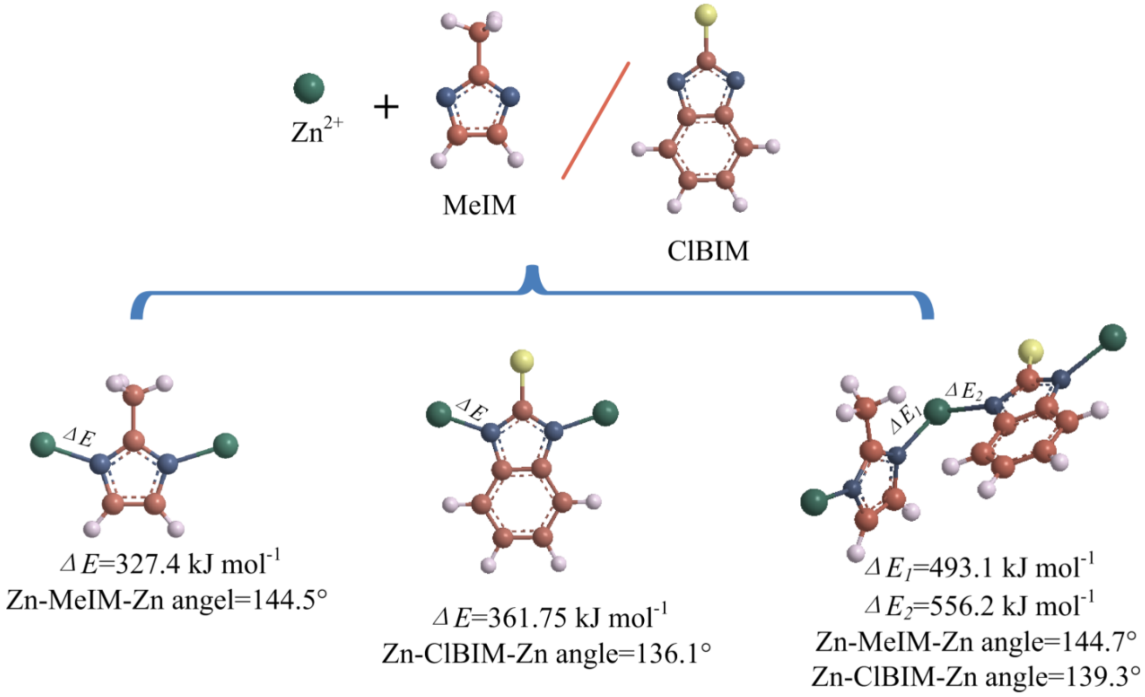

Figure S24. Bond energies and bond angles of Zn-linker-Zn.

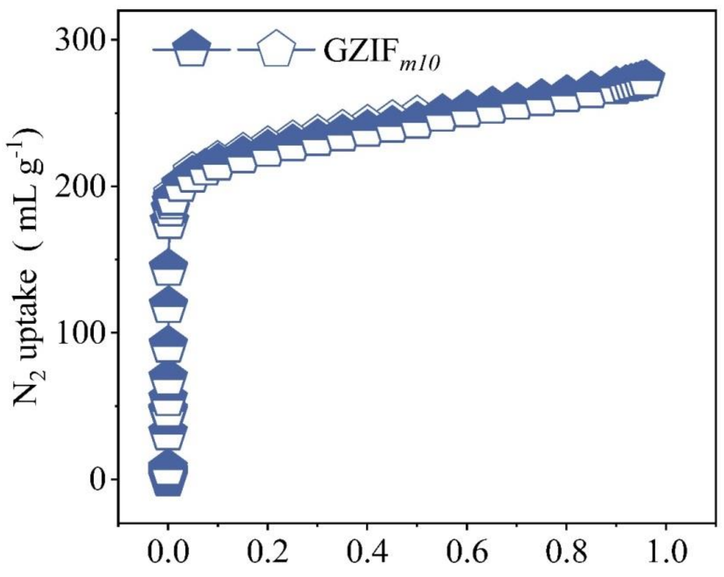

Figure S25. N_2 adsorption-desorption isotherms and pore width distributions of GZIF$_{m10}$.

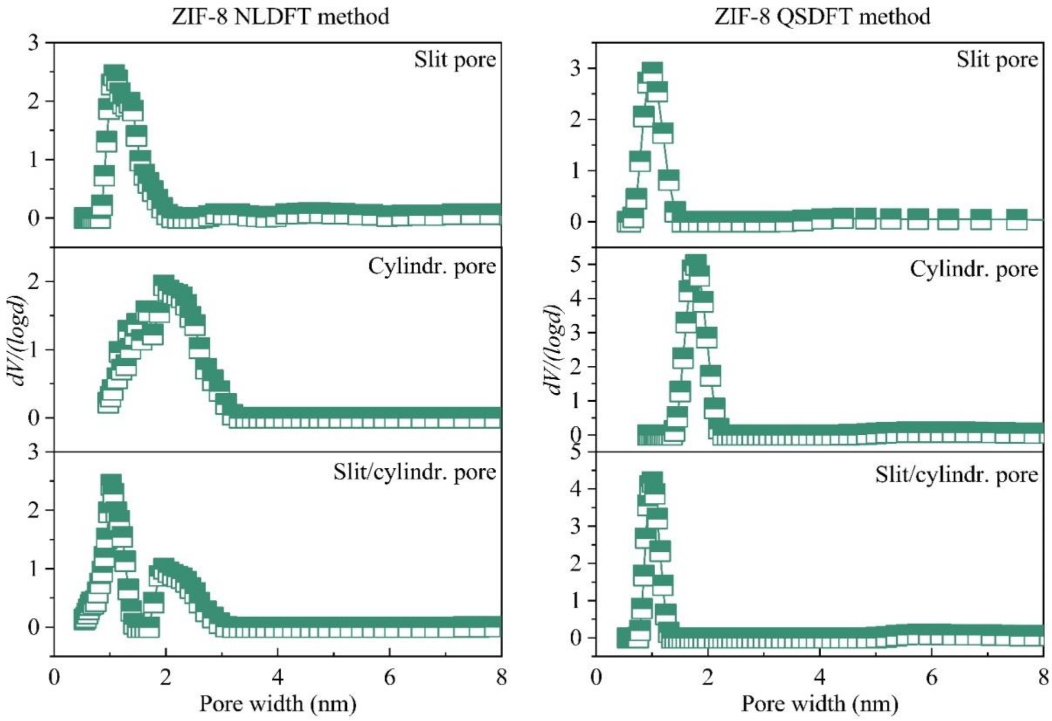

Figure S26. Pore width distribution curves of ZIF-8 calculated by NLDFT and QSDFT based on different pore models.

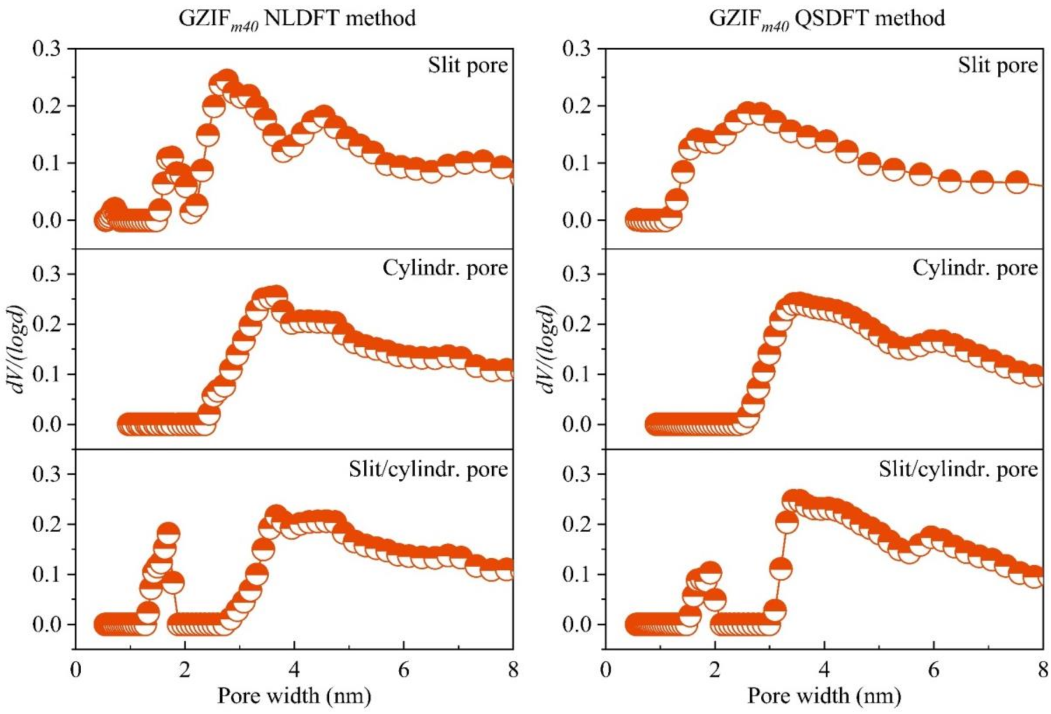

Figure S27. Pore width distribution curves of GZIF$_{m40}$ calculated by NLDFT and QSDFT based on different pore models.

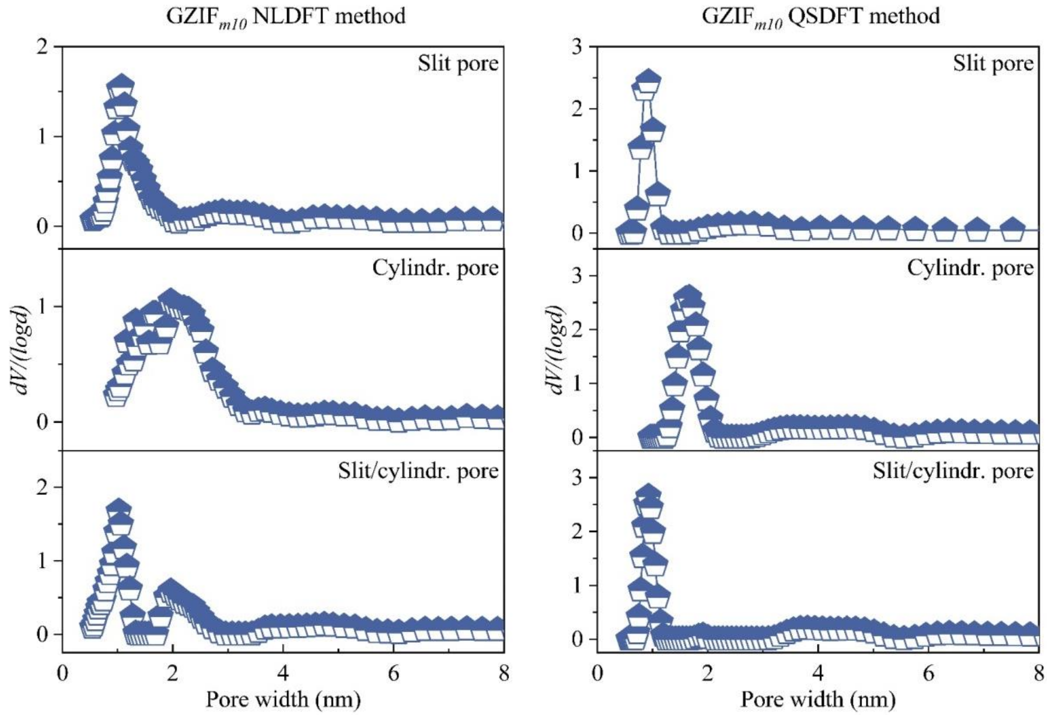

Figure S28. Pore width distribution curves of GZIF$_{m10}$ calculated by NLDFT and QSDFT based on different pore models.

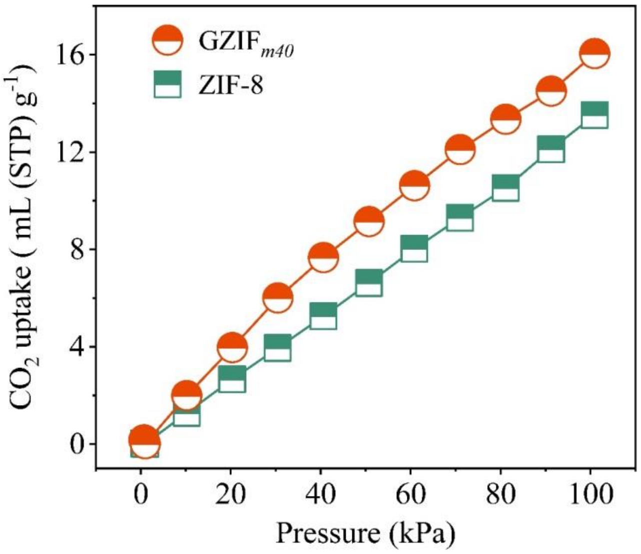

Figure S29. CO_2 isotherms of ZIF-8 and GZIF$_{m40}$ at 25 ^oC.

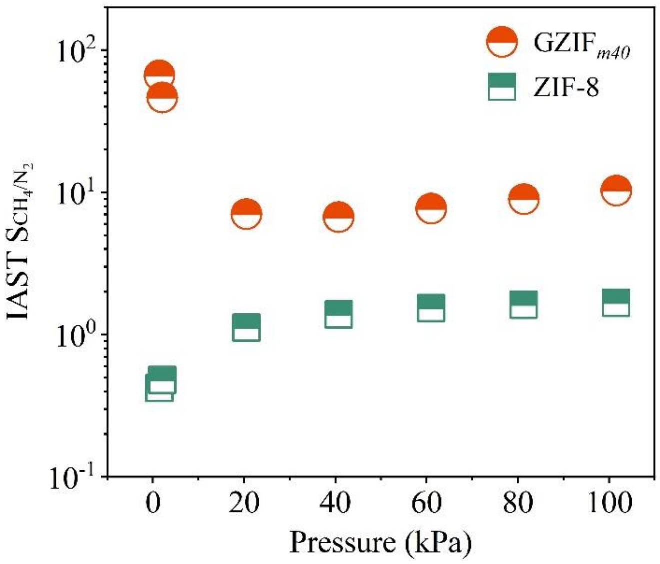

Figure S30. IAST selectivity of ZIF-8 and GZIF$_{m40}$ for the CH_4/N_2 (50:50) system.

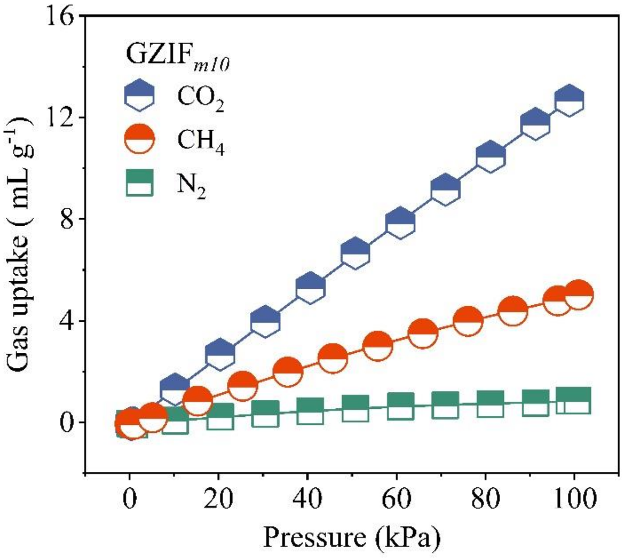

Figure S31. CO_2, CH_4, and N_2 sorption isotherms of GZIF$_{m10}$ at 25 °C.

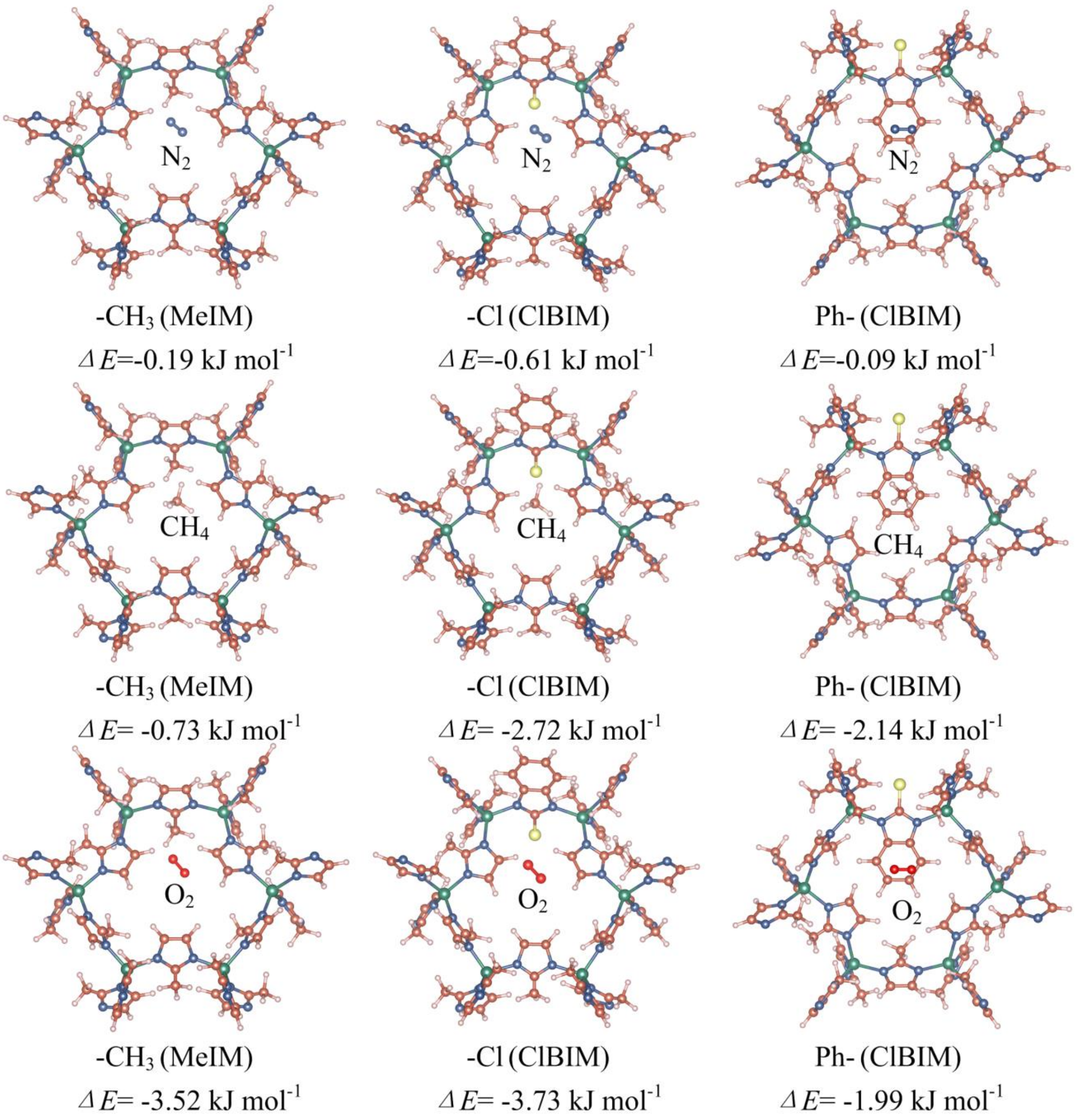

Figure S32. Binding energies of MeIM methyl in ZIF-8 and ClBIM chlorine and phenyl in GZIF$_m$ to N_2, CH_4, and O_2.

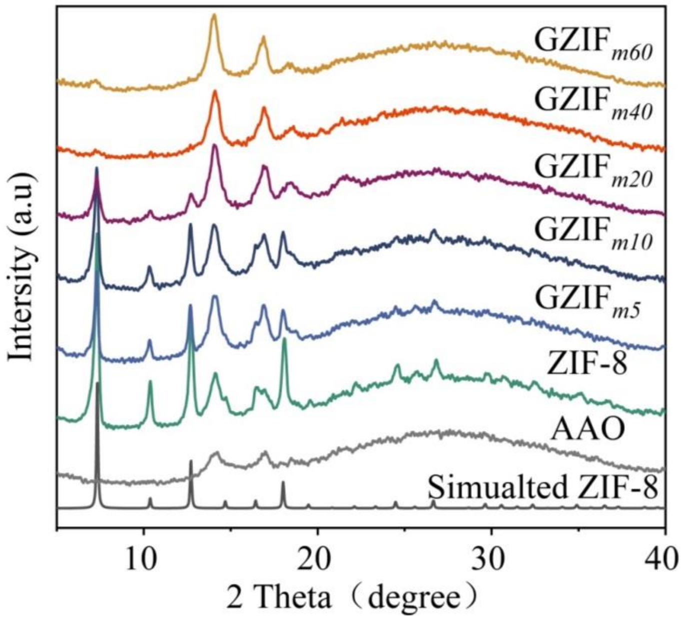

Figure S33. XRD patterns of the ZIF-8 and GZIF$_m$ membranes supported by the AAO substrates. XRD patterns of simulated ZIF-8 and AAO are presented for comparison.

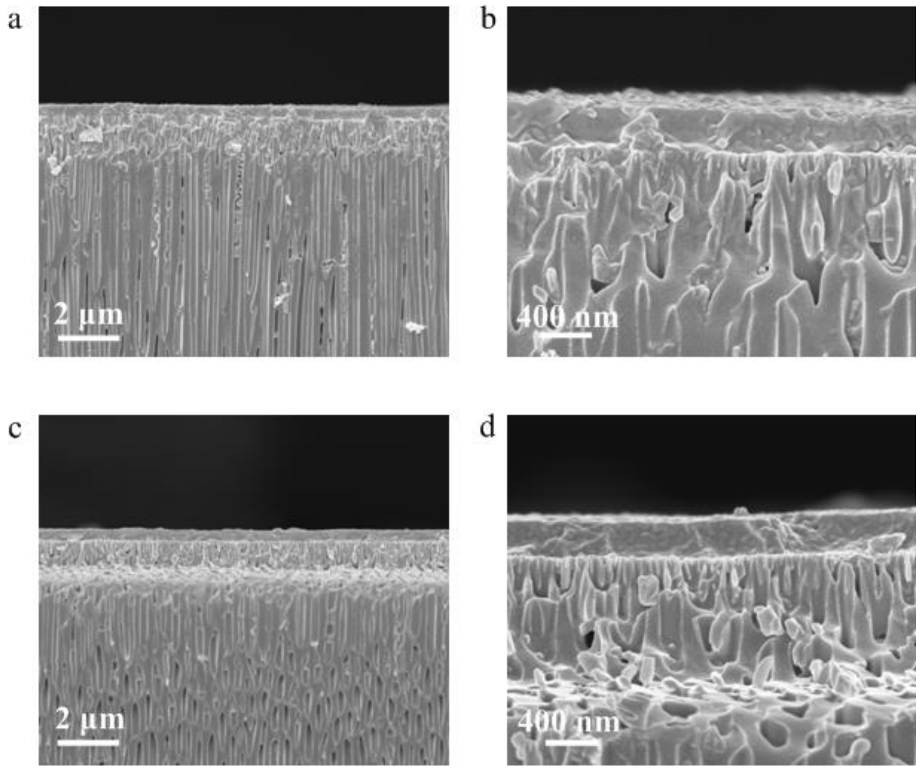

Figure S34. Cross-sectional SEM images of the ZIF-8 membrane. (a,b) sample 1. (c,d) sample 2.

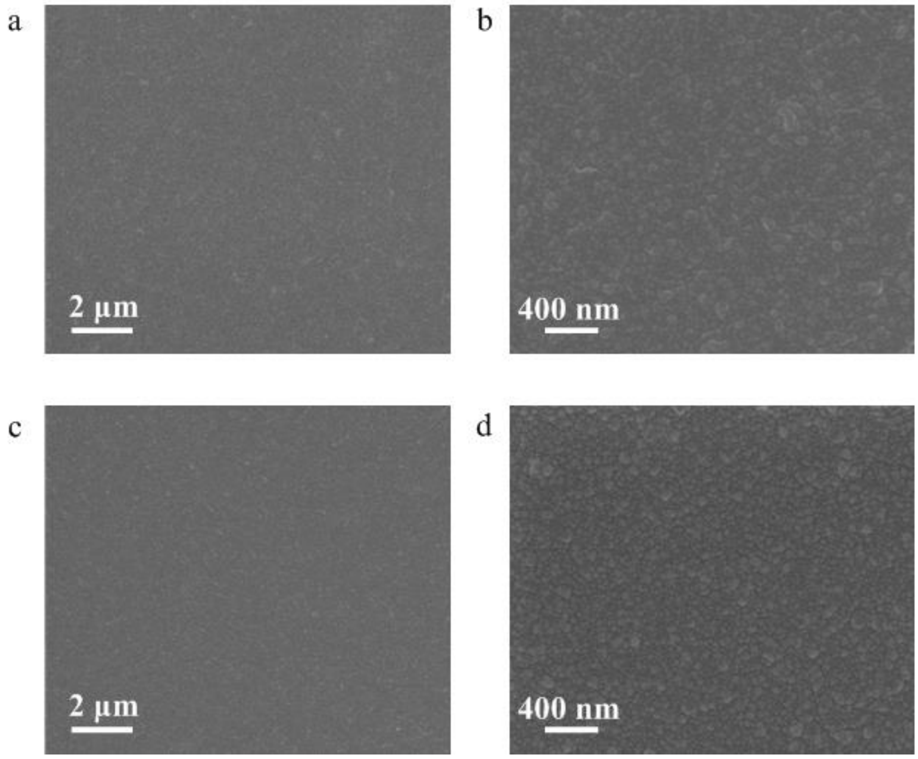

Figure S35. Top view SEM images of the ZIF-8 membrane. (a,b) sample 1. (c,d) sample 2.

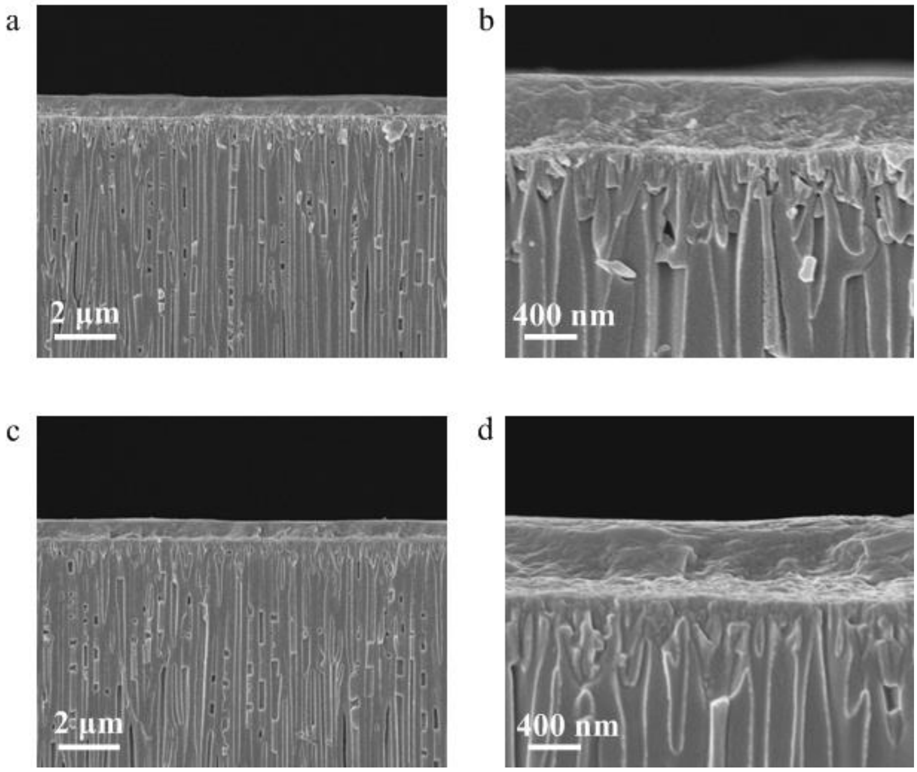

Figure S36 Cross-sectional SEM images of the $GZIF_{m40}$ membrane. (a,b) sample 1. (c,d) sample 2.

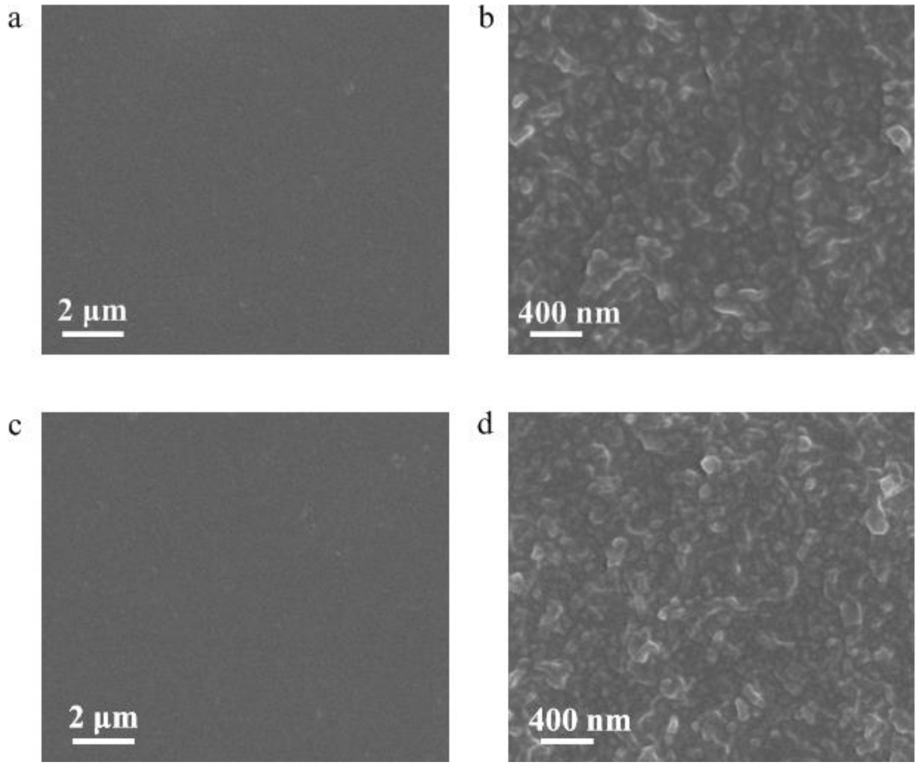

Figure S37. Top view SEM images of the $GZIF_{m40}$ membrane. (a,b) sample 1. (c,d) sample 2.

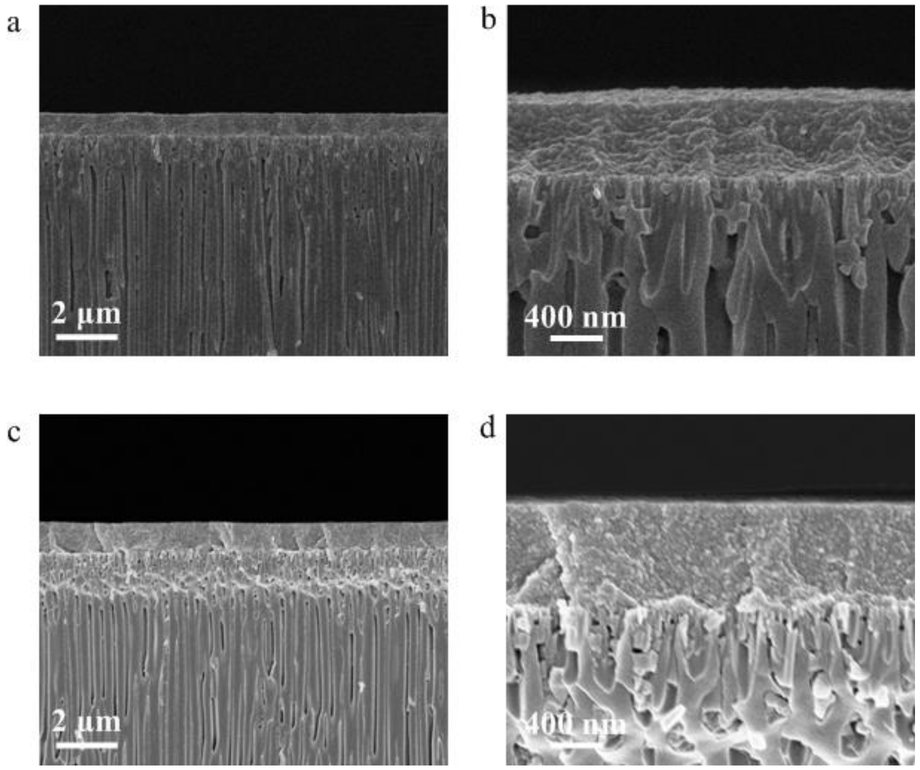

Figure S38. Cross-sectional SEM images of the $GZIF_m$ membranes. (a,b) $GZIF_{m10}$. (c,d) $GZIF_{m20}$.

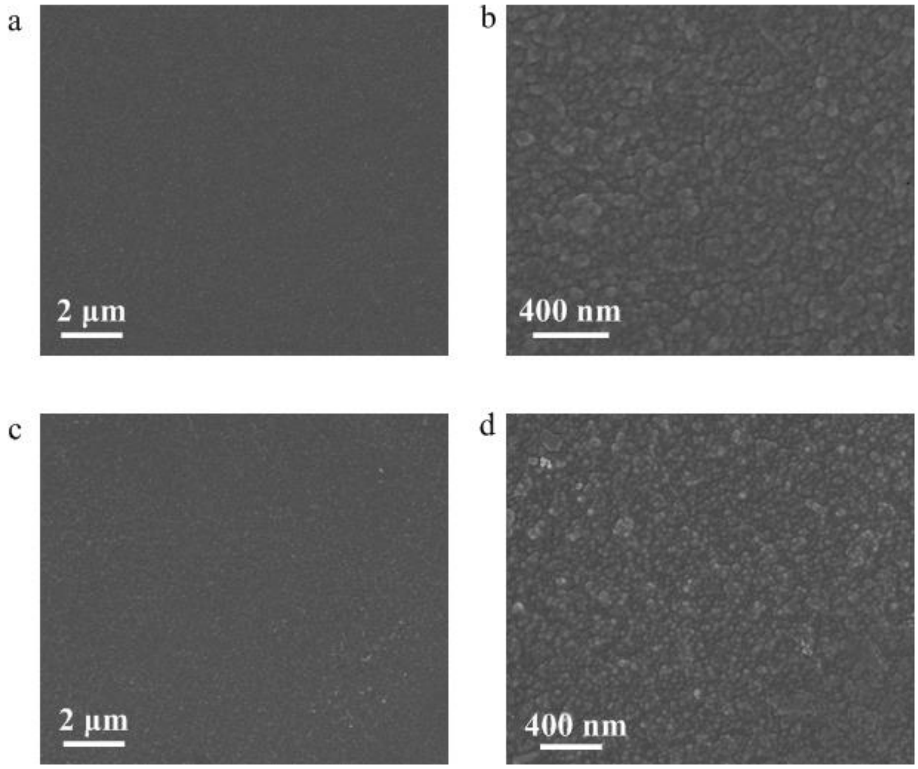

Figure S39. Top view SEM images of the $GZIF_m$ membranes. (a,b) $GZIF_{m10}$. (c,d) $GZIF_{m20}$.

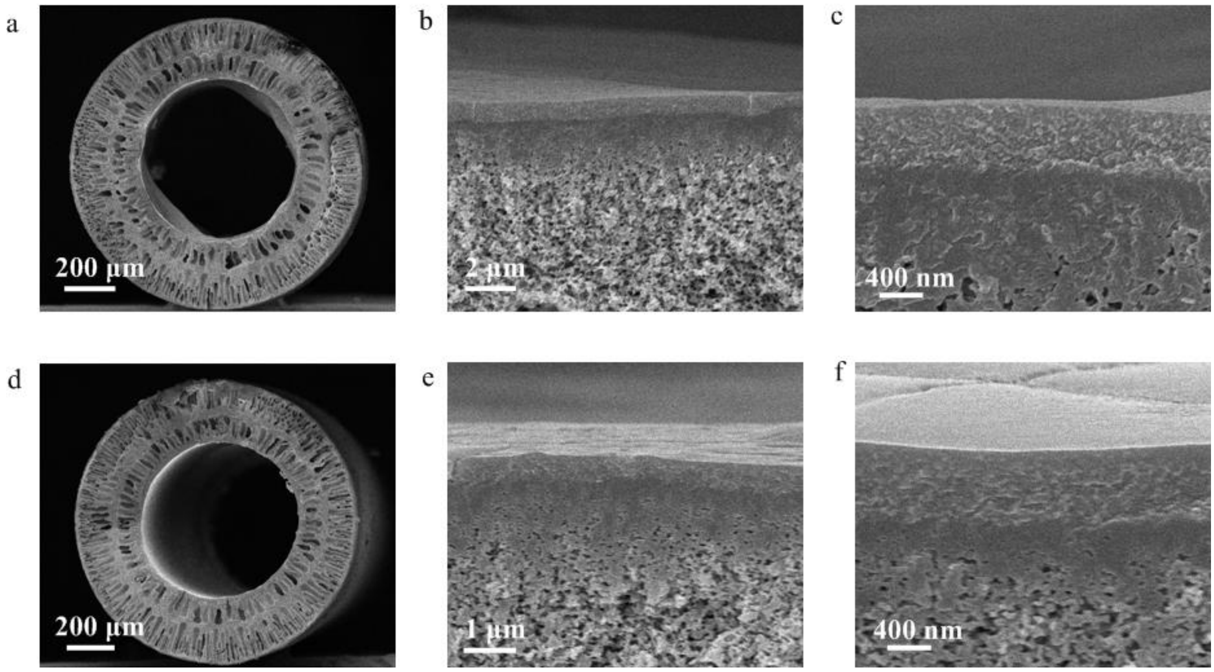

Figure S40. Cross-sectional SEM images of the ZIF-8 and $GZIF_m$ membranes supported by the polyvinylidene fluoride hollow fiber substrates. (a–c) ZIF-8. (d–f) $GZIF_m$.

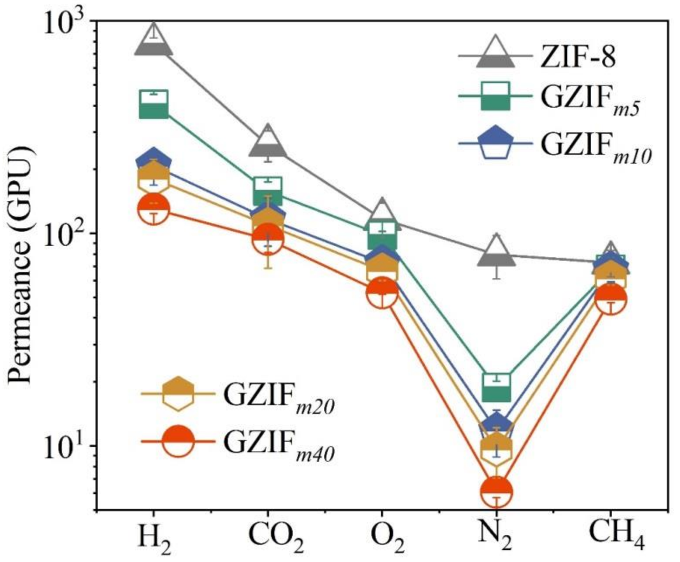

Figure S41. Single gas permeation properties of the ZIF-8 and GZIF$_m$ membranes.

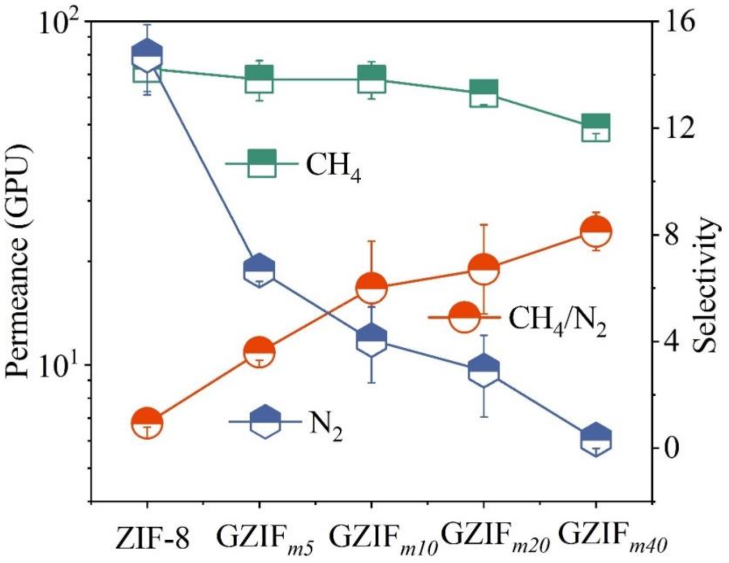

Figure S42. CH_4 permeance and CH_4/N_2 selectivity of the ZIF-8 and GZIF$_m$ membranes measured by single gas permeation.

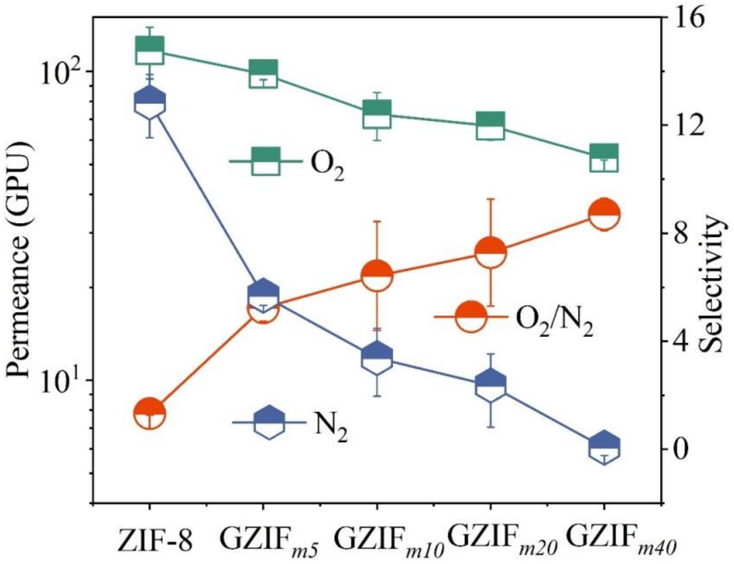

Figure S43. O_2 permeance and O_2/N_2 selectivity of the ZIF-8 and GZIF$_m$ membranes measured by single gas permeation.

Table S1. Summaries of fragility index, ΔC_p, and Young's modulus of MOF glass and MOF crystal-glass composites materials. a_g: amorphous glass, LDA: low-density amorphous, HDA: high-density amorphous, Im: imidazolate, GIS: gismondine, mbIm: 5-methylbenzimidazolate, 6-Cl-5-FbIm: 6-chloro-5-fluorobenzimidazolate, 5-Cl-2-mbIm: 5-chloro-2methylbenzimidazolate, 5-FbIm: 5-fluorobenzimidazolate, 5-ClbIm: 5chlorobenzimidazolate, IL: ionic liquid, HT: high temperature, DCI: 4,5-dicyanoimidazole, CN: 4-cynanoimidazolate, dCN: 4,5-dicyanoimidazolate, adp: adipate, mim: 2-methylimidazolate, bim: benzimidazolate, SSZ-13: aluminosilicate zeolites, and SAPO-34: silicoaluminophosphate zeolites.

Material	Fragility index	Heat capacity change ΔC_p (J g^{-1} K^{-1})	Young's modulus (GPa)	Ref
MOF glass				
a_gZIF-4 (LDA)	/	0.11	/	19
a_gZIF-4 (HDA)	/	0.16	/	19
a_gZIF-4	39	/	8.20	1
a_gTIF-4	23	/	7.90	1
a_gZIF-62	35	/	6.10 (710K)<br>8.80 (845K)	1
a_gZn(Im)$_2$ (GIS)	17	/	8.50	1
a_gZIF-62	23	0.19	/	20
a_gZIF-76	/	/	6.29	2
a_gZIF-76-mbIm	/	/	6.12	2
a_gZn(Im)$_{1.90}$(6-Cl-5-FbIm)$_{0.10}$	/	/	~8.00	15
a_gZn(Im)$_{1.75}$(5-Cl-2-	/	/	6.66	15

mbIm)$_{0.25}$				
$a_gZn(Im)_{1.63}(5\text{-}FbIm)_{0.37}$	/	/	9.40	15
$a_gZn(Im)_{1.69}(5\text{-}ClbIm)_{0.31}$	/	/	9.30	15
a_g(IL@ZIF-8-HT)	/	0.11	5.42	21
Zn-DCI-glass	/	/	126	16
Cd-DCI-glass	/	/	24	16
a_gZIF-4-CN$_{0.20}$	32.1	/	/	22
a_gZIF-4-dCN$_{0.18}$	32.8	/	/	22
a_gZIF-4-dCN$_{0.28}$	32.2	/	/	22
GMg-adp	33.0	/	18.29	14
a_gZIF-8-mim$_{0.15}$im$_{0.74}$bim$_{0.11}$	21.5	0.12	/	23
MOF crystal-glass composites				
(ZIF-4-Co)$_{0.5}$(ZIF-62)$_{0.5}$	/	/	7.50	24
(MIL-53)$_{0.25}$(a_gZIF-62)$_{0.75}$	/	/	7.70	17
(UiO-66)$_{0.25}$(a_gZIF-62)$_{0.75}$	/	/	7.90	17
($CsPbI_3$)$_{0.25}$(a_gZIF-62)$_{0.75}$	/	/	4.55	25
a_g[(ZIF-62)$_{0.9}$/(ZIF-8)$_{0.1}$]	/	/	5.49	3
a_g[(ZIF-62)$_{0.8}$/(ZIF-8)$_{0.2}$]	/	/	7.79	3
a_g[(ZIF-62)$_{0.7}$/(ZIF-8)$_{0.3}$]	/	/	7.67	3
a_g[(ZIF-62)$_{0.6}$/(ZIF-8)$_{0.4}$]	/	/	5.29	3
a_g[(ZIF-62)$_{0.5}$/(ZIF-8)$_{0.5}$]	/	/	4.16	3
a_g(ZIF-62)$_{0.9}$/(SSZ-13)$_{0.1}$	/	/	7.69	26
a_g(ZIF-62)$_{0.8}$/(SSZ-13)$_{0.2}$	/	/	11.58	26
a_g(ZIF-62)$_{0.7}$/(SSZ-13)$_{0.3}$	/	/	11.06	26

$a_g(ZIF\text{-}62)_{0.9}/(SAPO\text{-}34)_{0.1}$	/	/	8.03	26
$a_g(ZIF\text{-}62)_{0.8}/(SAPO\text{-}34)_{0.2}$	/	/	9.77	26
$a_g(ZIF\text{-}62)_{0.7}/ (SAPO\text{-}34)_{0.3}$	/	/	7.95	26
ZIF-8 (crystal)	/	/	3.26	This
$GZIF_{m40}$	25.4	0.19	17.50	work

Table S2. Porous properties of ZIF-8, $GZIF_{m10}$, and $GZIF_{m40}$.

Sample	BET surface area ($m^2\ g^{-1}$)	Microporous surface area ($m^2\ g^{-1}$)	Mesoporous surface area ($m^2\ g^{-1}$)
ZIF-8	1495	1406	89.3
$GZIF_{m10}$	865.2	741.4	123.8
$GZIF_{m40}$	114.8	25.1	89.7

Table S3. Performance summary of polymer, MOF, and mixed matrix membranes for CH_4/N_2 separation. Pebax: poly(ether-b-amide), Parel: primarily poly(propylene oxide), SBS: poly(styrene-b-butadiene-b-styrene), Tl: toluene, PMHS: Poly(methylhydrosiloxane), PDMS: Poly(dimethylsiloxane), SIS: poly(styrene-b-isoprene-b-styrene), PVAm: olyvinyl amine, PAA: polyacrylic acid, SBA: amine-functionalized in SBS, and PEI: polyethyleneimine. For membranes using permeability (Barrer), thicknesses are listed to calculate permeance (GPU).

Membranes	Ratio/Loading	Thickness (μm)	Permeance (GPU)	Selectivity	Ref
Pebax-2533	-	-	53.0	3.30	27
Parel-58	-	-	67.0	3.50	27
PIM-1	-	199	5.83	1.90	28
SBS21Tl	21 wt%	70	0.59	7.20	29
Pebax/SBS	10 wt%	90	0.48	2.80	30
SBS-c-PMHS	45 wt%	50	2.36	3.00	31
SIS-c-PDMS-co-PMHS	70 wt%	80	6.29	3.10	32
PVAm-CACl-10 (180)/MPSf	80 wt%	1	1648	3.10	33
PAA/-ZIF8@VR-600	60 wt%	0.6	987	2.67	34
PAA/ZIF-8@VR-800	67 wt%	0.8	1763	3.12	34
BAR-NAP-Benzene_CF$_3$@SBS*	-	-	~1×10^{-4}	9.54	35
SBA-15-NH_2/SBS	5 wt%	72.8	0.27	4.80	36
	10 wt%	84.3	0.28	7.30	36
Ni-MOF-74/SBS	15 wt%	70	1.07	2.30	37
[$Ni_3(HCOO)_6$]/SBS	5 wt%	77.5	1.00	2.90	38

ZIF-62-Br/PEI	54.5 wt%	-	2235	4.20	39
ZIF-7/AAO	-	1.5	35.4	1.07	40
HKUST-1/AAO	-	-	767.2	1.29	41
ZIF-8	-	-	76.6	0.95	This work
$GZIF_{m10}$	18.9%	-	64.9	5.94	This work
$GZIF_{m20}$	33.0%	-	60.2	7.36	This work
$GZIF_{m40}$	37.1%	-	51.6	9.29	This work

*The performance data of virtual membranes were calculated by computational simulations.

Table S4. Performance summary of polymer, MOF, and mixed matrix membranes for O_2/N_2 separation. PSF: polysulfone, CMS: carbon molecular sieve, PVP: poly(vinyl pyrrolidone), BADBSBF: 2,7-Bis(diamino-2',7'-di-tert-butyl-9,9'-spirobifluorene), BTDA: 3,32',7'-di-tert-butyl-tetracarboxylic dianhydride, PIM-1: polymer of intrinsic microporosity-1, ODPA: 4,4′-oxydiphthalic anhydride, TMDPA: 2,4,6-trimethyl-1,3-phenylenediamine, and CA: cellulose acetate. For membranes using permeability (Barrer), thicknesses are listed to calculate permeance (GPU).

Membranes	Ratio/Loading	Thickness (μm)	Permeance (GPU)	Selectivity	Ref
PSF-CMS30	30 wt%	60	0.12	3.69	42
PSF–PVP-sized CMS	30 wt%	60	0.11	6.05	42
BADBSBF-BTDA	-	30	0.60	9.00	43
PIM-1/silica	13 wt%	35	66.6	2.70	44
	19 wt%	35	92.1	2.10	44
	23.5 wt%	35	107	2.10	44
PIM-1/ZIF-71	10 wt%	60	13.4	3.60	45
	20 wt%	60	18.4	3.70	45
	30 wt%	60	26.7	3.50	45
MIL-101-PSF	24 wt%	60	0.11	5.80	46
MOF-5/Matrimid	0 wt%	35	0.05	7.60	47
	10 wt%	35	0.07	8.40	47
	20 wt%	35	0.08	7.20	47
	30 wt%	35	0.12	7.90	47
PIM-1/ZIF-8-6	-	70	16.3	3.30	48
PIM-1/ZIF-8-7	-	70	18.4	3.70	48

ODPA-TMDPA	-	71.5	0.14	5.20	49
ZIF-8/CA	5 wt %	57	0.06	9.70	50
ZSM-5/Matrimid	20 wt %	27	0.07	10.3	51
	30 wt %	29	0.12	8.50	51
ZIF-8	-	-	91.5	2.70	52
ZIF-8	-	-	113	1.28	This work
$GZIF_{m10}$	18.9%	-	71.5	6.55	This work
$GZIF_{m20}$	33.0%	-	68.7	8.10	This work
$GZIF_{m40}$	37.1%	-	55.6	9.57	This work

References

[1] T. D. Bennett, Y. Yue, P. Li, A. Qiao, H. Tao, N. G. Greaves, T. Richards, G. I. Lampronti, S, A. T. Redfern, F. Blanc, O. K. Farha, J. T. Hupp, A. K. Cheetham, D. A. Keen, *J. Am. Chem. Soc.* **2016**, *138*, 3484-3492.

[2] S. Li, R. Limbach, L. Longley, A. A. Shirzadi, J. C. Walmsley, D. N. Johnstone, P. A. Midgley, L. Wondraczek, T. D. Bennett, *J. Am. Chem. Soc.* **2019**, *141*, 1027-1034.

[3] D. Ao, Z. Yang, Z. Qiao, Y. Sun, Z. Zhang, M. D. Guiver, C. Zhong, *Angew. Chem. Int. Ed.* **2023**, *62*, e202304535.

[4] F. Q. Yuan, C. Z. Cai, S. Zhao, *Explo. Shock.* **2013**, *1*, 79-84.

[5] L. Song, W. Liu, F. Xin, Y. Li, *Int. J. Adhes.* **2021**, *106*, 102820.

[6] J. P. Perdew, K. Burke, M. Ernzerhof, *Phys. Rev. Lett.* **1996**, *77*, 3865.

[7] D. Vanderbilt, *Phys. Rev. B.* **1990**, *41*, 7892.

[8] Y. Hu, Z. Liu, J. Xu, Y. Huang, Y. Song, *J. Am. Chem. Soc.* **2013**, *135*, 9287-9290.

[9] H. Zhang, M. Zhao, Y. Yang, Y. S. Lin, *Microporous Mesoporous Mater.* **2019**, *288*, 109568.

[10] J. Liang, B. Johannessen, Z. Wu, R. F. Webster, J. Yong, M. Y. B. Zulkifli, J. S. Harbort, Y. R. Cheok, H. Wen, Z. Ao, B. Kong, S. L. Y. Chang, J. Scott, K. Liang, *Adv. Mater.* **2022**, *34*, 2205674.

[11] C. Yu, X. Cen, Z. Zhang, Y. Sun, W. Xue, Z. Qiao, M. D. Guiver, C. Zhong, *Adv. Mater.* **2023**, *35*, 2307013.

[12] F. Zhou, Y. Chen, Z. Zhang, Z. Gu, Y. Sun, M. Tong, Q. Yang, W. Dong, Y. Pan, Z. Qiao, C. Zhong, *AIChE J.* **2024**, *70*, e18455.

[13] Z. Huang, J. Rath, Q. Zhou, A. Cherevan, S. Naghdi, D. Eder, *Small* **2024**, *20*, 2307981.

[14] M. Kim, H. S. Lee, D. H. Seo, S. J. Cho, E. Jeon, H. R. Moon, *Nat. Commun.* **2024**, *15*,

1174.

[15] J. Hou, M. L. Ríos Gómez, A. Krajnc, A. McCaul, S. Li, A. M. Bumstead, A. F. Sapnik, Z. Deng, R. Lin, P. A. Chater, D. S. Keeble, D. A. Keen, D. Appadoo, B. Chan, V. Chen, G. Mali, T. D. Bennett, *J. Am. Chem. Soc.* **2020**, *142*, 3880-3890.

[16] B. Zhou, Z. Qi, D. Yan, *Angew. Chem. Int. Ed.* **2022**, *61*, e202208735.

[17] J. Hou, C. W. Ashling, S. M. Collins, A. Krajnc, C. Zhou, L. Longley, D. N. Johnstone, P. A. Chater, S. Li, M. V. Coulet, P. L. Llewellyn, F. X. Coudert, D. A. Keen, P. A. Midgley, G. Mali, V. Chen, T. D. Bennett, *Nat. Commun.* **2019**, *10*, 2580.

[18] X. Ma, W. Higgins, Z. Liang, D. Zhao, G. M. Pharr, K. Y. Xie, *Proc. Natl. Acad. Sci. U. S. A.* **2021**, *118*, e2025657118.

[19] T. D. Bennett, J. C. Tan, Y. Yue, E. Baxter, C. Ducati, N. J. Terrill, H. H. M. Yeung, Z. Zhou, W. Chen, S. Henke, A. K. Cheetham, G. N. Greaves, *Nat. Commun.* **2015**, *6*, 8079.

[20] A. Qiao, T. D. Bennett, H. Tao, A. Krajnc, G. Mali, C. M. Doherty, A. W. Thornton, J. C. Mauro, G. N. Greaves, Y. Yue, *Sci. Adv.* **2018**, *4*, eaao6827.

[21] V. Nozari, C. Calahoo, J. M. Tuffnell, D. A. Keen, T. D. Bennett, L. Wondraczek, *Nat. Commun.* **2021**, *12*, 5703.

[22] J. Song, L. Frentzel-Beyme, R. Pallach, P. Kolodzeiski, A. Koutsianos, W. L. Xue, R. Schmid, S. Henke, *J. Am. Chem. Soc.* **2023**, *145*, 9273-9284.

[23] W. L. Xue, P. Kolodzeiski, H. Aucharova, S. Vasa, A. Koutsianos, R. Pallach, J. Song, L. Frentzel-Beyme, R. Linser, S. Henke, *Nat. Commun.* **2024**, *15*, 4420.

[24] L. Longley, S. M. Collins, C. Zhou, G. J. Smales, S. E. Norman, N. J. Brownbill, C. W. Ashling, P. A. Chater, R. Tovey, C. B. Schönlieb, T. F. Headen, N. J. Terrill, Y. Yue, A. J. Smith, F. Blanc, D. A. Keen, P. A. Midgley, T. D. Bennett, *Nat. Commun.* **2018**, *9*, 2135.

[25] J. Hou, P. Chen, A. Shukla, A. Krajnc, T. Wang, X. Li, R. Doasa, L. H. Tizei, B. Chan, D. N. Johnstone, R. Lin, T. U. Schülli, I. Martens, D. Appadoo, M. S' Ari, Z. Wang, T. Wei, S. C. Lo, M. Lu, S. Li, E. B. Namdas, G. Mali, A. K. Cheetham, S. M. Collins, V. Chen, L.

Wang, T. D. Bennett, *Science* **2021**, *374*, 621-625.

[26] D. Ao, Z. Yang, A. Chen, Y. Sun, M. Ye, L. Tian, X. Cen, Z. Xie, J. Du, Z. Qiao, A. K. Cheetham, J. Hou, C. Zhong, *Angew. Chem. Int. Ed.* **2024**, *63*, e202401118.

[27] K. A. Lokhandwala, I. Pinnau, Z. He, K. D. Amo, A. R. DaCosta, J. G. Wijmans, R. W Baker, *J. Membr. Sci.* **2010**, *346*, 270-279.

[28] P. M. Budd, N. B. McKeown, B. S. Ghanem, K. J. Msayib, D. Fritsch, L. Starannikova, N. Belov, O. Sanfirova, Y. Yampolskii, V. Shantarovich, *J. Membr. Sci.* **2008**, *325*, 851-860.

[29] M. G. Buonomenna, G. Golemme, C. M. Tone, M. P. De Santo, F. Ciuchi, E. Perrotta, *Adv. Funct. Mater.* **2012**, *22*, 1759-1767.

[30] P. Guan, J. Luo, W. Li, Z. Si, *Macromol. Res.* **2017**, *25*, 1007-1014.

[31] J. Luo, T. Zhu, Y. Song, Z. Si, *Polymer* **2017**, *127*, 52-65.

[32] X. Yang, Y. Zheng, L. Wang, Q. Guo, H. Shan, Z. Xu, J. Luo, *React. Funct. Polym.* **2019**, *142*, 36-43.

[33] L. Guan, Z. Ma, X. Guo, Z. Qiao, C. Zhong, *Chem. Eur. J.* **2021**, *16*, 3236-3243.

[34] Z. Gu, Z. Yang, X. Guo, Z. Qiao, C. Zhong, *Sep. Purif. Technol.* **2021**, *272*, 118845.

[35] T. Yan, D. Liu, Q. Yang, C. Zhong, *Chin. J. Chem. Eng.* **2022**, *42*, 170-177.

[36] M. G. Buonomenna, G. Golemme, C. M. Tone, M. P. De Santo, F. Ciuchi, E. Perrotta, *J. Mater. Chem. A* **2013**, *1*, 11853-11866.

[37] S. Wang, Q. Guo, S. Liang, P. Li, J. Luo, *Sep. Purif. Technol.* **2018**, *199*, 206-213.

[38] S. Wang, Q. Guo, S. Liang, P. Li, X. Li, J. Luo, *Chem. Eng. Technol.* **2018**, *41*, 353-366.

[39] C. Ma, N. Li, W. Xue, X. Guo, Z. Qiao, C. Zhong, *J. Membr. Sci.* **2023**, *683*, 121829.

[40] Y. Li, Y. Liang, H. Bux, A. Feldhoff, W. Yang, J. Caro, *Angew. Chem. Int. Ed.* **2010**, *49*, 548-551.

[41] J. Nan, X. Dong, W. Wang, W. Jin, N. Xu, *Langmuir* **2011**, *27*, 4309-4312.

[42] W. A. W. Rafizah, A. F. Ismail, *J. Membr. Sci.* **2008**, *307*, 53-61.

[43] Y. H. Kim, H. S. Kim, S. K. Kwon, *Macromolecules* **2005**, *38*, 7950-7956.

[44] J. Ahn, W. Chung, I. Pinnau, J. Song, N. Du, G. P. Robertson, M. D. Guiver, *J. Membr. Sci.* **2010**, *346*, 280-287.

[45] L. Hao, K. S. Liao, T. S. Chung, *J. Mater. Chem. A* **2015**, *3*, 17273-17281.

[46] H. B. Jeazet, C. Staudt, C. Janiak, *Chem. Commun.* **2012**, *48*, 2140-2142.

[47] E. V. Perez, K. J. Balkus, J. P. Ferraris, I. H. Musselman, *J. Membr. Sci.* **2009**, *328*, 165-173.

[48] Y. Liu, J. Zhang, X. Tan, *ACS omega* **2019**, *4*, 16572-16577.

[49] C. Duan, X. Jie, D. Liu, Y. Cao, Q. Yuan, *J. Membr. Sci.* **2014**, *466*, 92-102.

[50] S. U. Azam, A. Hussain, S. Farrukh, T. Noor, Y. Liu, *Environ. Sci. Pollut. Res.* **2020**, *27*, 24413-24429.

[51] Y. Zhang, K. J. Balkus, I. H. Musselman, J. P. Ferraris, *J. Membr. Sci.* **2008**, *325*, 28-39.

[52] N. Hara, M. Yoshimune, H. Negishi, K. Haraya, S. Hara, T. Yamaguchi, *J. Membr. Sci.* **2014**, *450*, 215-223.
